# Adverse Event Signals Associated with Beta-Lactamase Inhibitors: Disproportionality Analysis of USFDA Adverse Event Reporting System

**DOI:** 10.3390/jox15050144

**Published:** 2025-09-09

**Authors:** Kannan Sridharan, Gowri Sivaramakrishnan

**Affiliations:** 1 Department of Pharmacology & Therapeutics, College of Medicine & Medical Sciences, Arabian Gulf University, Manama 26671, Bahrain; 2 Bahrain Defence Force Royal Medical Services, Riffa 28743, Bahrain; gowri.sivaramakrishnan@gmail.com

**Keywords:** avibactam, clavulanic acid, tazobactam, relebactam, sulbactam, vaborbactam

## Abstract

Background: Beta-lactamase inhibitors (BLIs) are widely used with beta-lactam antibiotics to combat resistant infections, yet their safety profiles, especially for newer agents, remain underexplored. This study aimed to identify potential adverse event (AE) signals associated with BLIs using the USFDA Adverse Event Reporting System (USFDA AERS). Methods: The USFDA AERS was queried for AE reports involving FDA-approved BLIs from March 2004 to March 2024. After removing duplicates, only reports with BLIs listed as primary suspects were included. Disproportionality analysis was conducted using frequentist and Bayesian approaches, with statistical significance assessed by chi-square testing. Results: A total of 12,456 unique reports were analyzed. Common AEs across BLIs included hematologic disorders, hypersensitivity reactions, emergent infections, organ dysfunction, and neurological complications. Signal detection revealed specific associations: septic shock and respiratory failure with avibactam; lymphadenopathy and congenital anomalies with clavulanic acid; antimicrobial resistance and epilepsy with relebactam; disseminated intravascular coagulation and cardiac arrest with sulbactam; and agranulocytosis and conduction abnormalities with tazobactam. For vaborbactam, no distinct AE signals were identified apart from off-label use. Mortality was significantly more frequent with avibactam and relebactam (*p* < 0.0001). Conclusions: This analysis highlights a spectrum of AE signals with BLIs, including unexpected associations warranting further investigation. While some events may reflect comorbidities or concomitant therapies, these findings underscore the importance of continued pharmacovigilance and targeted clinical studies to clarify causality and ensure the safe use of BLIs in practice.

## 1. Introduction

Beta-lactam antimicrobials (penicillins, carbapenems, cephalosporins, and monobactams) constitute a vital class of antibiotics within the antimicrobial arsenal. These agents are widely preferred due to their broad antimicrobial spectrum, favorable safety profile, and tolerable adverse effects. However, the extensive use of β-lactam antibiotics has been observed to primarily drive the production of β-lactamases that hydrolyze the β-lactam ring, leading to the development of resistance amongst both Gram-positive and Gram-negative pathogens [1]. To date, sequence analysis has identified over 8200 distinct β-lactamase enzymes [2].

To combat this resistance, β-lactamase inhibitors (BLIs) have been developed and are commonly co-administered with β-lactam antibiotics to enhance their efficacy against drug-resistant infections. Currently, six BLIs, avibactam, clavulanic acid, relebactam, sulbactam, tazobactam, and vaborbactam, are approved by the United States Food and Drug Administration (USFDA) [3]. Clavulanic acid, sulbactam, and tazobactam were developed earlier and were initially effective against a limited range of β-lactamase enzymes. However, the rise of extended-spectrum β-lactamases and carbapenemases necessitated the development of novel BLIs such as avibactam, relebactam, and vaborbactam, which exhibit enhanced activity against these resistant strains [4]. These newer BLIs are particularly valuable in managing infections caused by multidrug-resistant *Enterobacterales* and *Pseudomonas aeruginosa* [5].

The first-generation BLIs, clavulanic acid, sulbactam, and tazobactam, are β-lactam-based molecules structurally related to penicillins; they act as “suicide substrates” by forming a covalent acyl-enzyme complex with serine β-lactamases [6]. In contrast, the more recently approved BLIs represent structural innovations designed to expand inhibitory activity. Avibactam and relebactam are diazabicyclooctane (DBO) derivatives, lacking the classical β-lactam core but instead possessing a bicyclic urea scaffold that provides reversible covalent inhibition of both class A and some class C and D β-lactamases [7]. Vaborbactam, on the other hand, belongs to the cyclic boronate class, with a boronic acid moiety that mimics the tetrahedral intermediate of β-lactam hydrolysis, conferring potent activity against class A carbapenemases, notably *Klebsiella pneumoniae* carbapenemase (KPC) [8].

Interestingly, some BLIs exhibit weak intrinsic antibacterial activity against specific pathogens. Sulbactam, for example, is active against *Acinetobacter spp.*, *Neisseria gonorrhoeae*, and *Bacteroides* spp.; clavulanic acid is effective against *Haemophilus influenzae* and *Neisseria gonorrhoeae*; and tazobactam shows activity against *Borrelia burgdorferi* [9,10,11].

Despite their widespread use, the adverse event profiles of BLIs are not comprehensively characterized. Reported adverse effects include gastrointestinal symptoms (nausea, constipation, and diarrhea), nervous system disorders (seizures and insomnia), altered platelet function, and hypersensitivity reactions, including anaphylaxis, Stevens–Johnson syndrome (SJS), toxic epidermal necrolysis (TEN), and eosinophilia [12]. The USFDA Adverse Event Reporting System (AERS) collects spontaneously reported adverse events from healthcare professionals and is publicly accessible [13]. Disproportionality analysis of the USFDA AERS database has proven to be a valuable tool in identifying potential adverse event signals, warranting further exploration through clinical studies or mechanistic models [14].

However, there is a significant lack of literature on the adverse event profiles of BLIs, particularly the novel agents. A recent disproportionality analysis of the USFDA AERS database identified 654 reports associated with ceftolozane–tazobactam and 506 reports related to ceftazidime–avibactam, revealing signals of agranulocytosis and encephalopathy, respectively [15]. Beyond this, no prior study has systematically evaluated the adverse event signals across the entire spectrum of FDA-approved BLIs. Therefore, the novelty of our work lies in providing the first comprehensive pharmacovigilance assessment of both early- and recently approved BLIs using the FAERS database, thereby addressing an important gap in safety characterization.

## 2. Methods

### 2.1. Data Source

We queried the USFDA AERS database using the names of the USFDA-approved beta-lactamase inhibitors listed in Table 1 [16]. The data analyzed in this study encompassed 81 quarterly reports spanning March 2004 (the earliest publicly available USFDA AERS dataset) to March 2024 (the most recent complete dataset at the time of analysis).

### 2.2. Data Processing

Data processing followed the USFDA’s recommendations for the removal of duplicate reports [17]. Cases were identified using their unique case identification numbers, and for each case, only the entry with the highest individual safety report number was retained, with duplicate records removed. The USFDA categorizes the association between drugs and suspected adverse events into four categories: primary suspect, secondary suspect, interacting, or concomitant. For the purposes of this study, only unique reports in which a BLI was designated as the primary suspect were included, as this classification reflects the reporter’s assessment of the most likely causal drug and reduces confounding from concomitant therapies. From each unique report, data on gender, year of report, adverse events, and outcomes were extracted. Missing data points within the reports were excluded from further analysis.

### 2.3. Data Mining Algorithms

For this disproportionality analysis, we applied the case–non-case methodology to identify potential safety signals. In this approach, the frequency of specific adverse events reported with the drug of interest (“cases”) is compared against the frequency of the same events reported with all other drugs (“non-cases”) [18]. Signal detection was performed using the OpenVigil 2.1 platform, which incorporates both frequentist and Bayesian statistical methods.

Within the frequentist framework, the measures assessed included the reporting odds ratio (ROR) and the proportional reporting ratio (PRR) [19]. The ROR was expressed with 95% confidence intervals (CIs). A signal was considered statistically significant if it satisfied the following thresholds: a minimum of three independent case reports, PRR of at least 2, chi-square (χ^2^) value of 4 or greater, and a lower 95% CI bound for the ROR exceeding 1 [20].

For the Bayesian approach, the Bayesian Confidence Propagation Neural Network (BCPNN) and Multi-item Gamma Poisson Shrinker (MGPS) methods were applied to estimate signal detection measures. In BCPNN, signals were identified when the lower 95% CI limit of the information component (IC025) exceeded zero. In the Empirical Bayes geometric mean (EBGM) model, a signal was considered significant when the lower 95% CI limit of EBGM05 exceeded 2 [20].

The reported outcomes were categorized into one of the following: death, life-threatening events, or hospitalization (initial or prolonged).

### 2.4. Statistical Analysis

The demographic characteristics were described using descriptive statistics. Categorical variables were summarized as percentages. The chi-square (χ^2^) test was applied to evaluate the significance of differences in the distribution of reported outcomes. A *p*-value ≤ 0.05 was interpreted as statistically significant. All statistical analyses were performed using SPSS software (IBM Corp., Armonk, NY, USA; Version 27.0, released 2020).

### 2.5. Search Results

**Figure 1:**
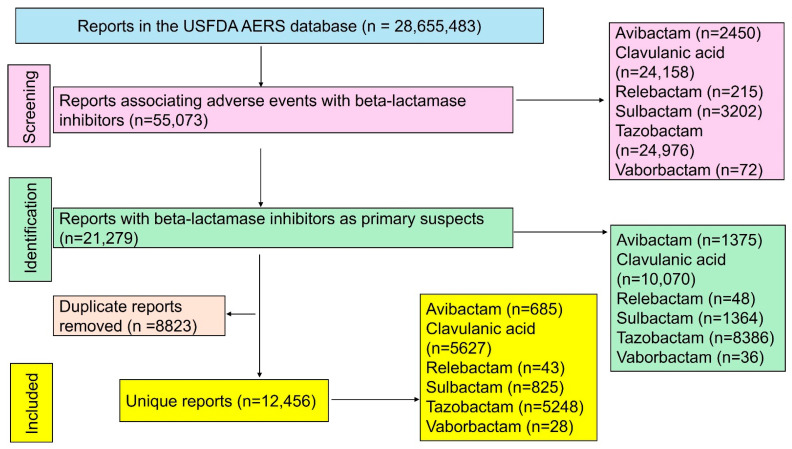
Study flow diagram. A total of 12,456 unique reports were included in the final analysis.

Out of the 28,655,483 reports that were available in the USFDA AERS database, 12,456 unique reports were included in the final analysis (Figure 1). Clavulanic acid (n = 5627) and tazobactam (n = 5248) accounted for most reports, followed by other beta-lactamase inhibitors. The demographic characteristics of patients associated with each beta-lactamase inhibitor are summarized in Table 2. Excluding unreported data, most patients were elderly (>65 years) and predominantly male.

### 2.6. Signals Detected for Beta-Lactamase Inhibitors

Common adverse events identified across all beta-lactamase inhibitors included hematologic disorders such as myelosuppression, hemolysis, thrombocytopenia, neutropenia, eosinophilia, and hemolytic anemia. Hypersensitivity reactions, including anaphylaxis, drug reaction with eosinophilia and systemic symptoms (DRESS), Stevens–Johnson syndrome (SJS), and toxic epidermal necrolysis (TEN), were also observed, along with increased susceptibility to infections such as *Clostridium difficile*, *Klebsiella*, and *Candida*. Additional adverse events included organ dysfunction, particularly altered hepatic function, renal failure, and multiple organ dysfunction syndrome, as well as neurological disorders like encephalopathy, epilepsy, neurotoxicity, and delirium.

Specific adverse events associated with individual beta-lactamase inhibitors were as follows:

- Avibactam: Reported adverse events included eosinophilia, myelosuppression, death, septic shock, osteomyelitis, hyponatremia, neurotoxicity, encephalopathy, delirium, and respiratory failure (Table 3).
- Clavulanic Acid: Associated with lymphadenopathy, splenomegaly, congenital anomalies, hypergammaglobulinemia, Kounis syndrome, mastoiditis, conjunctivitis, diarrhea, oral candidiasis, enterocolitis, megacolon, and serum-sickness-like reaction (Table 4).
- Relebactam: Primarily showed signals of antimicrobial resistance and epilepsy (Table 5).
- Sulbactam: Notable adverse events included disseminated intravascular coagulation, hemorrhagic diathesis, Kounis syndrome, cardiogenic shock, cardiac arrest, prolonged activated partial thromboplastin time, anaphylactic reaction, elevated creatine phosphokinase, and coagulopathy (Table 6).
- Tazobactam: Specific adverse events included agranulocytosis, lymphohistiocytosis, prolonged prothrombin time, Evans syndrome, platelet dysfunction, cardiac conduction abnormalities, pulseless electrical activity, anaphylactic reaction, and perinatal complications (Table 7).
- Vaborbactam: No specific adverse events were reported, apart from off-label use (Table 8).

### 2.7. Comparison of Outcomes Between Adverse Events with Beta-Lactamase Inhibitors

**Figure 2:**
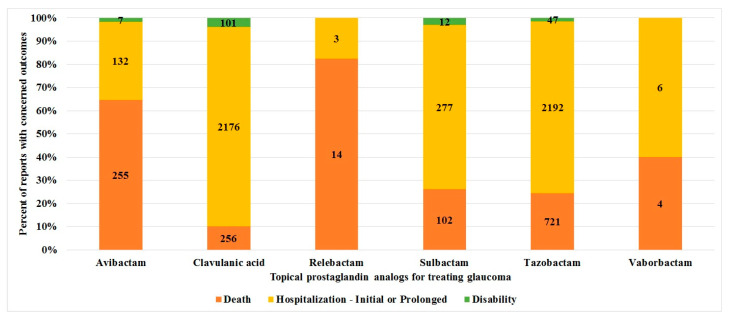
Comparison of the reported outcomes between beta-lactamase inhibitors. The stacked bar chart depicts the distribution of key outcomes reported for each beta-lactamase inhibitor.

The distribution of key outcomes among beta-lactamase inhibitors is presented in Figure 2. Death was reported more frequently with avibactam and relebactam compared to other inhibitors, with a statistically significant difference (*p* < 0.0001).

## 3. Discussion

### 3.1. Key Findings of This Study

Common adverse events reported for all beta-lactamase inhibitors included hematologic disorders (myelosuppression, hemolysis, thrombocytopenia), hypersensitivity reactions (anaphylaxis, DRESS, SJS, TEN), risk of emergent infections (Clostridium difficile, Klebsiella, Candida), organ dysfunction (hepatic, renal, multiple organ), and neurological issues (encephalopathy, epilepsy, delirium). Avibactam-specific events include septic shock and respiratory failure, while clavulanic acid shows lymphadenopathy, congenital anomalies, and enterocolitis. Relebactam is linked with antimicrobial resistance and epilepsy. Sulbactam shows disseminated intravascular coagulation and cardiac arrest, and tazobactam is associated with agranulocytosis and cardiac conduction abnormalities. Vaborbactam only reported off-label use.

### 3.2. Comparison with the Existing Literature

Among the adverse event signals identified in this study, hematologic reactions, including myelosuppression and hemolysis, were unexpected. However, these could be attributed to concomitant diseases or drugs causing myelotoxicity, particularly since tazobactam combinations are frequently used in patients with hematological malignancies [21]. Similarly, renal failure, hepatic failure, and multiple organ dysfunction, although unexpected, may result from underlying conditions such as sepsis or the use of other concurrent medications. Discontinuation of beta-lactam/BLI combinations due to organ failure has been previously reported [22]. The emergence of infections, particularly iatrogenic infections like Clostridium difficile, has also been documented with broad-spectrum β-lactam/BLIs [23]. Notably, the incidence of such infections appears higher with newer generations of cephalosporins and tetracyclines, whose usage has declined with the introduction of β-lactam/BLIs due to their comparable efficacy and spectrum [24].

Congenital anomalies associated with clavulanic acid use were identified as potential signals in this study. Clavulanic acid is commonly co-administered with amoxicillin, and epidemiological data have suggested an increased risk of cleft lip with/without cleft palate (odds ratio of 2), particularly when used in the last trimester of pregnancy (odds ratio of 4.3) [25]. However, a prospective, controlled study involving 191 pregnant women exposed to amoxicillin–clavulanic acid during the first trimester found no significant increase in the risk of major congenital malformations (1.9% with amoxicillin–clavulanic acid versus 3% in controls) [26]. Therefore, the association of clavulanic acid with congenital anomalies observed in this study is likely a false signal.

The adverse event patterns observed in this analysis can be interpreted in the context of the distinct chemical scaffolds and mechanisms of action of different BLIs. The classical β-lactam-based inhibitors such as clavulanic acid, sulbactam, and tazobactam share structural similarity with penicillins, which may explain the strong association with hypersensitivity reactions, cutaneous eruptions, and hepatic cholestasis seen in the disproportionality signals, reflecting immunoallergic pathways commonly linked to β-lactam cores. In contrast, the newer DBO derivatives, avibactam and relebactam, which lack a β-lactam ring, demonstrated signals of neurotoxicity, encephalopathy, epilepsy, and septic shock, which may be attributable to their broader β-lactamase binding spectrum and reversible covalent inhibition mechanism, raising the possibility of off-target interactions in neuronal or metabolic pathways. Vaborbactam, a cyclic boronate inhibitor, showed no distinct adverse event signals apart from off-label use, which may reflect its novel boronic-acid-based scaffold and limited clinical exposure to date [27]. Interestingly, signals of hematologic toxicity such as agranulocytosis with tazobactam and myelosuppression with avibactam further suggest potential structural contributions to bone marrow suppression. Similarly, the identification of congenital anomalies and lymphadenopathy with clavulanic acid could be linked to its β-lactam structural reactivity and potential immune-modulatory effects. Taken together, these findings highlight how the evolution of BLI structures, from β-lactam analogues to DBOs and cyclic boronates, may contribute to differing adverse event profiles, underlining the importance of considering both chemical class and mechanism of action when interpreting pharmacovigilance signals.

### 3.3. Strengths and Limitations

This study leverages a large dataset from the USFDA AERS, offering a comprehensive evaluation of adverse event signals associated with beta-lactamase inhibitors. A major strength of this analysis is the use of robust statistical methods, including frequentist and Bayesian approaches, which enhance the reliability of signal detection. However, the study has several limitations. First, the USFDA AERS is based on spontaneous reporting, which may be subject to underreporting, reporting biases, and incomplete data, potentially affecting the accuracy of the findings. Additionally, the study’s observational nature precludes establishing causality between the drugs and the adverse events. The lack of detailed clinical information in the reports, such as concomitant medications, underlying comorbidities, and dosing regimens, limits the ability to fully understand the context of the adverse events. Furthermore, novel BLIs such as vaborbactam had limited data due to their recent approval, restricting the analysis of adverse events associated with these newer agents.

Moving forward, prospective studies with more detailed clinical data and controlled settings are essential to validate these findings and establish causality. Expanding pharmacovigilance efforts and enhancing reporting accuracy through better healthcare provider awareness and electronic health record integration could provide more precise adverse event data. Additionally, mechanistic studies exploring the pathophysiology of unexpected adverse events, particularly hematologic and organ dysfunctions, are warranted to better understand the safety profile of BLIs and inform safer clinical use.

## 4. Conclusions

This study highlights a broad spectrum of adverse events associated with beta-lactamase inhibitors, including hematologic, hypersensitivity, infectious, organ dysfunction, and neurological disorders. While some adverse events are consistent with known safety profiles, unexpected signals such as myelosuppression, hemolysis, and organ dysfunction were also observed, warranting further investigation. The findings underscore the importance of continuous pharmacovigilance and signal detection to monitor the safety of both established and novel BLIs. Given the limitations of spontaneous reporting systems, future research should focus on prospective studies and mechanistic evaluations to better understand the causality and clinical implications of these adverse events. This knowledge is critical for optimizing the safe use of beta-lactamase inhibitors in clinical practice, particularly in populations with complex comorbidities.

## Figures and Tables

**Table 1:** USFDA approved beta-lactamase inhibitors.

Beta-Lactamase Inhibitors	Year of USFDA Approval
Avibactam	2015
Clavulanic acid	1984
Relebactam	2019
Sulbactam	1986
Tazobactam	2005
Vaborbactam	2017

**Table 2:** Demographic characteristics of patients in the reports.

Characteristics		Avibactam (n = 685)	Clavulanic Acid (n = 5627)	Relebactam (n = 43)	Sulbactam (n = 825)	Tazobactam (n = 5248)	Vaborbactam (n = 28)
Age groups [n (%)]	<18	16 (2.3)	641 (11.4)	0	54 (6.5)	277 (5.3)	0
	18 to <45	20 (2.9)	1066 (18.9)	3 (7)	130 (15.8)	791 (15.1)	5 (17.9)
	45 to <65	63 (9.2)	1158 (20.6)	12 (27.9)	141 (17.1)	1360 (25.9)	5 (17.9)
	≥65	69 (10.1)	1354 (24.1)	13 (30.2)	308 (37.3)	1938 (36.9)	5 (17.9)
	Not specified	517 (75.5)	1408 (25)	15 (34.9)	192 (23.3)	882 (16.8)	13 (46.3)
Quantitative age (years)	Mean (SD)	57.5 (23.4)	49.2 (25.3)	64.4 (15.2)	56.7 (24.3)	58 (22.1)	53.7 (16.4)
	Median (range)	61 (1–95)	53 (1–103)	63.5 (25–87)	64 (0–100)	62 (1–101)	61 (26–83)
Gender [n (%)]	Male	310 (45.3)	2121 (37.7)	27 (62.8)	371 (45)	2803 (53.4)	11 (39.3)
	Female	158 (23.1)	2851 (50.7)	16 (37.2)	275 (33.3)	1893 (36.1)	5 (17.9)
	Unknown	217 (31.7)	655 (11.6)	0	179 (22.7)	552 (10.5)	12 (42.9)
Reporting top countries		USA, UK, GE, CN	USA, UK, FR, CA	USA, JP	USA, UK, JP	USA, FR, CA, JP	USA, GE

USA: The United States of America; UK: The United Kingdom; FR: France; GE: Germany; CN: China; CA: Canada.; and JP: Japan.

**Table 3:** Summary of signal detection measures for reports associated with avibactam.

SOC	MedDRA Code	Adverse Event	PRR	χ^2^	RRR	ROR [95% CI]	IC025	EBGM05	Number of Cases
Blood and lymphatic system disorders	10014950	Eosinophilia	8.7	20.1	8.7	8.7 [3.3, 23.4]	1.2	3.3	4
	10028584	Myelosuppression	5.2	9.8	5.2	5.3 [2, 14]	0.9	2	4
	10018910	Hemolysis	15.6	27.7	15.6	15.7 [5, 48.7]	1.3	5	3
Cardiac disorders	10007515	Cardiac arrest	3.3	10.4	3.3	3.3 [1.6, 6.6]	0.8	1.6	8
Gastrointestinal disorders	10082129	Dysbiosis	387.8	2279.5	379.8	391.7 [184.6, 831.3]	4	179	7
	10027141	Melena	4.7	5.5	4.7	4.8 [1.5, 14.8]	0.7	1.5	3
General disorders and administration site conditions	10011906	Death	4.1	317.8	4.1	4.8 [4, 5.8]	4	18.7	131
	10059866	Drug resistance	41.9	1355.2	41.8	44.1 [31.4, 62]	3.8	29.7	35
	10010264	Condition aggravated	2.0	8.0	2.0	2.1 [1.3, 3.3]	0.6	1.3	17
	10077361	Multiple organ dysfunction syndrome	20.0	233.5	19.9	20.4 [12, 34.6]	2.5	11.7	14
	10066901	Treatment failure	5.3	44.6	5.3	5.4 [3.2, 9.1]	1.4	3.1	14
	10051118	Drug ineffective for unapproved indication	5.3	27.1	5.3	5.3 [2.8, 10.3]	1.2	2.7	9
	10061819	Disease recurrence	3.8	5.6	3.8	3.8 [1.4, 10.1]	0.7	1.4	4
Hepatobiliary disorders	10019670	Hepatic function abnormal	8.6	53.5	8.6	8.8 [4.5, 16.9]	1.6	4.5	9
	10072268	Drug-induced liver injury	9.3	51.1	9.3	9.4 [4.7, 18.8]	1.6	4.6	8
	10060795	Hepatic enzyme increased	4.3	17.1	4.3	4.3 [2.2, 8.7]	1	2.1	8
	10008635	Cholestasis	10.5	34.1	10.5	10.6 [4.4, 25.6]	1.4	4.4	5
	10019837	Hepatocellular injury	11.4	28.1	11.3	11.4 [4.3, 30.5]	1.3	4.2	4
	10067125	Liver injury	6.1	12.2	6.1	6.1 [2.3, 16.3]	1	2.3	4
Infections and infestations	10034133	Pathogen resistance	156.6	6903.3	155.3	167.8 [124.3, 226.7]	5.4	115	46
	10040070	Septic shock	10.2	82.5	10.2	10.4 [5.7, 18.8]	1.8	5.6	11
	10061259	Klebsiella infection	74.1	575.9	73.8	75.1 [38.8, 145.1]	3.2	38.2	9
	10040047	Sepsis	3.1	10.5	3.1	3.1 [1.6, 5.9]	0.8	1.6	9
	10061471	Pseudomonas infection	39.2	260.0	39.1	39.6 [19.7, 79.6]	2.6	19.4	8
	10074170	Candida infection	7.6	11.1	7.6	7.6 [2.4, 23.6]	0.9	1.4	3
	10054236	Clostridium difficile infection	6.3	8.7	6.3	6.3 [2, 19.7]	0.9	2	3
	10031252	Osteomyelitis	6.1	8.3	6.1	6.2 [2, 19.1]	0.8	2	3
	10035717	Pneumonia Klebsiella	68.1	136.4	67.8	68.4 [21.9, 212.9]	2	21.8	3
Injury, poisoning, and procedural complications	10053762	Off-label use	4.2	263.9	4.2	4.8 [3.9, 5.9]	1.7	3.4	105
	10076309	Product use issue	3.4	27.7	3.4	3.4 [2.1, 5.5]	1.1	2.1	18
	10079843	Product storage error	2.7	7.3	2.7	2.8 [1.4, 5.6]	0.7	1.4	8
	10081581	Incorrect product administration duration	6.4	13.3	6.4	6.4 [2.4, 17.2]	1	2.4	4
	10081578	Product administered to patient of inappropriate age	16.5	29.6	16.5	16.6 [5.3, 51.6]	1.3	5.3	3
Investigations	10035528	Platelet count decreased	5.0	44.5	5.0	5.1 [3.1, 8.5]	1.4	3	15
	10005483	Blood creatinine increased	7.1	62.3	7.1	7.2 [4.2, 12.5]	1.6	4.1	13
	10047942	White blood cell count decreased	3.2	13.1	3.2	3.2 [1.7, 6.1]	0.9	1.7	10
	10043554	Thrombocytopenia	2.6	6.7	2.6	2.7 [1.3, 5.4]	0.7	1.3	8
	10005364	Blood bilirubin increased	7.9	29.5	7.9	7.9 [3.6, 17.7]	1.3	3.5	6
	10033318	Oxygen saturation decreased	3.9	8.0	3.9	3.9 [1.6, 9.4]	0.8	1.6	5
	10054889	Transaminases increased	7.9	23.4	7.8	7.9 [3.3, 19]	1.2	3.3	5
	10005802	Blood sodium decreased	8.0	17.9	8.0	8.0 [3, 21.4]	1.1	3	4
	10004685	Bilirubin conjugated increased	48.8	96.6	48.7	49.1 [15.8, 152.7]	1.8	15.6	3
	10059570	Blood alkaline phosphatase increased	4.5	5.0	4.5	4.5 [1.4, 13.9]	0.7	1.4	3
	10017693	Gamma-glutamyl transferase increased	5.4	6.7	5.4	5.4 [1.7, 16.7]	0.8	1.7	3
	10051608	Platelet count increased	7.8	11.7	7.8	7.8 [2.5, 24.4]	1	2.5	3
Nervous system disorders	10029350	Neurotoxicity	20.9	169.8	20.9	21.2 [11.3, 39.5]	2.3	11.2	10
	10014625	Encephalopathy	13.0	87.7	12.9	13.1 [6.8, 25.3]	1.9	6.7	9
	10012373	Depressed level of consciousness	6.6	32.5	6.6	6.6 [3.3, 13.3]	1.4	3.3	8
	10015037	Epilepsy	8.9	48.9	8.9	9.0 [4.5, 18.2]	1.6	4.5	8
	10039906	Seizure	2.4	5.2	2.4	2.4 [1.2, 4.8]	0.6	1.2	8
	10010071	Coma	4.7	16.8	4.7	4.7 [2.2, 9.9]	1.1	2.2	7
	10001854	Altered state of consciousness	8.4	25.4	8.3	8.4 [3.5, 20.3]	1.3	3.4	5
	10029202	Nervous system disorder	4.3	9.7	4.3	4.4 [1.8, 10.5]	0.9	1.8	5
	10028622	Myoclonus	7.9	11.9	7.9	8.0 [2.6, 24.8]	1	2.5	3
	10034759	Petit mal epilepsy	19.8	36.4	19.8	19.9 [6.4, 61.9]	1.4	6.4	3
Psychiatric disorders	10012218	Delirium	8.1	43.1	8.1	8.2 [4.1, 16.5]	1.5	4	8
Renal and urinary disorders	10038435	Renal failure	3.7	28.7	3.7	3.7 [2.3, 6.1]	1.1	2.2	16
	10062237	Renal impairment	4.8	32.1	4.8	4.8 [2.7, 8.5]	1.3	2.7	12
	10069339	Acute kidney injury	2.4	8.1	2.4	2.5 [1.4, 4.5]	0.7	1.3	11
Respiratory, thoracic, and mediastinal disorders	10038695	Respiratory failure	4.0	14.9	4.0	4.0 [2, 8]	1	2	8
	10043089	Tachypnea	7.9	11.8	7.9	7.9 [2.6, 24.7]	1	2.5	3
Skin and subcutaneous tissue disorders	10073508	Drug reaction with eosinophilia and systemic symptoms	4.9	8.9	4.9	4.9 [1.9, 13.2]	0.9	1.8	4
	10044223	Toxic epidermal necrolysis	6.7	9.5	6.7	6.8 [2.2, 21]	0.9	2.2	3

SOC: System organ classification; MedDRA: Medical dictionary of adverse events; PRR: Proportional reporting ratio; χ^2^: Chi-square test; RRR: Relative reporting ratio; ROR: Reporting odds ratio; IC: Information component; and EBGM: Empirical Bayes Geometric Mean.

**Table 4:** Summary of signal detection measures for reports associated with clavulanic acid.

SOC	MedDRA	Adverse Event	PRR	χ^2^	RRR	ROR [95% CI]	IC025	EBGM05	Number of Reports
Blood and lymphatic system disorders	10025197	Lymphadenopathy	4.3	83.0	4.3	4.3 [3.1, 6.1]	1.5	3.1	34
	10041660	Splenomegaly	3.7	17.0	3.7	3.7 [2, 6.9]	1	2	10
	10029783	Normochromic normocytic anemia	9.5	37.3	9.5	9.5 [4.3, 21.2]	1.5	4.2	6
	10020973	Hypocoagulable state	19.2	51.4	19.0	19.2 [7.2, 51.3]	1.6	7.1	4
	10055128	Autoimmune neutropenia	40.6	78.2	39.9	40.6 [13, 127.3]	1.7	12.7	3
	10020630	Hypergammaglobulinemia	30.8	58.4	30.4	30.8 [9.9, 96.2]	1.6	9.7	3
	10025188	Lymphadenitis	6.2	8.5	6.2	6.2 [2, 19.4]	0.8	2	3
	10025226	Lymphangitis	16.8	29.9	16.6	16.8 [5.4, 52.2]	1.3	5.3	3
Cardiac disorders	10069167	Kounis syndrome	85.8	3874.9	82.7	86.5 [65, 115.3]	4.8	62.1	49
	10028606	Myocarditis	2.7	6.0	2.7	2.7 [1.3, 5.7]	0.7	1.3	7
	10074636	Fetal heart rate deceleration abnormality	17.0	30.3	16.9	17.0 [5.5, 52.9]	1.3	5.4	3
Congenital, familial, and genetic disorders	10025391	Macroglossia	22.8	42.1	22.6	22.8 [7.3, 71.1]	1.4	7.2	3
	10084407	Otospondylomegaepiphyseal dysplasia	259.2	479.6	232.5	259.4 [78.5, 857.2]	2.4	70.4	3
Ear and labyrinth disorders	10014025	Ear swelling	9.1	21.2	9.1	9.1 [3.4, 24.3]	1.2	3.4	4
	10026900	Mastoiditis	14.8	26.0	14.8	14.9 [4.8, 46.2]	1.2	4.7	3
Eye disorders	10015993	Eyelid edema	10.6	250.3	10.6	10.7 [7.4, 15.3]	2.4	7.4	30
	10015967	Eye swelling	2.7	22.6	2.7	2.7 [1.8, 4.1]	0.9	1.8	23
	10034545	Periorbital edema	15.8	274.1	15.7	15.8 [10.3, 24.3]	2.6	10.2	21
	10010741	Conjunctivitis	4.4	43.4	4.4	4.4 [2.8, 7]	1.3	3.7	18
	10058117	Ocular icterus	8.8	54.7	8.8	8.8 [4.6, 17]	1.6	4.6	9
	10051625	Conjunctival hyperemia	9.2	50.4	9.2	9.2 [4.6, 18.5]	1.6	4.6	8
	10056647	Periorbital swelling	3.6	5.1	3.6	3.6 [1.3, 9.5]	0.7	1.3	4
Gastrointestinal disorders	10012735	Diarrhea	3.3	774.0	3.3	3.5 [3.2, 3.8]	1.6	3	476
	10047700	Vomiting	3.0	429.2	3.0	3.1 [2.8, 3.5]	1.4	2.7	323
	10072268	Drug-induced liver injury	28.6	5231.9	28.2	29.6 [25.7, 34.1]	4.2	24.5	200
	10000081	Abdominal pain	2.5	121.3	2.5	2.6 [2.2, 3.1]	1.1	2.1	130
	10000087	Abdominal pain upper	2.4	90.3	2.4	2.4 [2, 2.9]	1	2	113
	10024558	Lip edema	65.1	3861.9	63.3	65.8 [51.2, 84.9]	4.7	49.3	64
	10024570	Lip swelling	5.8	182.7	5.8	5.9 [4.4, 7.8]	1.9	4.4	47
	10043967	Tongue edema	47.8	1884.0	46.8	48.2 [35.6, 65.2]	4	34.6	43
	10043528	Throat tightness	5.2	108.5	5.2	5.3 [3.7, 7.4]	1.7	3.7	33
	10016100	Feces discolored	5.5	95.3	5.5	5.5 [3.8, 8.1]	1.7	3.8	27
	10042727	Swollen tongue	3.3	39.6	3.3	3.3 [2.3, 4.9]	1.2	2.2	26
	10056998	Palatal oedema	78.0	1763.1	75.4	78.4 [52.5, 116.8]	4.2	50.6	25
	10016102	Feces pale	50.7	1096.0	49.6	50.9 [34, 76.4]	3.8	33.1	24
	10018836	Hematochezia	2.0	11.6	2.0	2.0 [1.4, 3]	0.7	1.4	24
	10044032	Tooth discoloration	32.0	591.0	31.5	32.1 [20.8, 49.4]	3.2	20.5	21
	10012741	Diarrhea hemorrhagic	9.4	133.7	9.4	9.4 [6, 14.8]	2.1	5.9	19
	10068318	Oropharyngeal discomfort	9.8	140.7	9.8	9.8 [6.3, 15.4]	2.1	6.2	19
	10033647	Pancreatitis acute	3.0	19.6	3.0	3.0 [1.9, 4.9]	1	1.8	16
	10034829	Pharyngeal edema	3.9	32.3	3.9	4.0 [2.4, 6.5]	1.2	2.4	16
	10082270	Pharyngeal swelling	7.3	74.9	7.3	7.3 [4.4, 12.1]	1.7	4.4	15
	10030094	Odynophagia	11.2	119.9	11.2	11.3 [6.7, 19]	2.1	6.6	14
	10061008	Biliary tract disorder	36.4	372.3	35.8	36.5 [20.6, 64.5]	2.9	20.2	12
	10030963	Oral candidiasis	4.8	32.3	4.8	4.8 [2.7, 8.5]	1.3	2.7	12
	10027141	Melena	2.1	5.4	2.1	2.1 [1.2, 3.8]	0.6	1.2	11
	10038776	Retching	2.3	7.0	2.3	2.3 [1.3, 4.2]	0.7	1.3	11
	10014893	Enterocolitis	8.2	55.9	8.2	8.2 [4.4, 15.3]	1.6	4.4	10
	10027110	Megacolon	28.8	237.9	28.4	28.8 [15.4, 53.8]	2.6	15.2	10
	10002959	Aphthous ulcer	6.0	32.5	6.0	6.0 [3.1, 11.6]	1.3	3.1	9
	10057009	Pharyngeal erythema	18.8	133.6	18.7	18.9 [9.8, 36.4]	2.2	9.7	9
	10082129	Dysbiosis	54.1	357.2	52.9	54.2 [26.9, 109.4]	2.8	26.2	8
	10014896	Enterocolitis hemorrhagic	21.6	136.1	21.4	21.7 [10.8, 43.5]	2.2	10.7	8
	10017999	Gastrointestinal pain	3.4	11.3	3.4	3.4 [1.7, 6.8]	0.9	1.7	8
	10080124	Lip erythema	36.5	170.6	36.0	36.6 [16.3, 82]	2.3	16	6
	10000097	Abdominal tenderness	4.4	10.1	4.4	4.4 [1.8, 10.7]	0.9	1.8	5
	10008417	Cheilitis	4.0	8.6	4.0	4.0 [1.7, 9.7]	0.8	1.7	5
	10051402	Dysentery	11.1	36.1	11.0	11.1 [4.6, 26.7]	1.4	4.6	5
	10030110	Edema mouth	5.5	14.1	5.5	5.5 [2.3, 13.2]	1	2.3	5
	10036772	Proctalgia	4.0	8.6	4.0	4.0 [1.7, 9.7]	0.8	1.7	5
	10042101	Stomach discomfort	2.9	4.6	2.9	2.9 [1.2, 7]	0.6	1.2	5
	10048946	Anal abscess	4.1	6.4	4.1	4.1 [1.5, 10.8]	0.8	1.5	4
	10036200	Portal hypertension	5.5	10.7	5.5	5.5 [2.1, 14.8]	0.9	2.1	4
	10050247	Anal inflammation	26.9	50.4	26.5	26.9 [8.6, 83.9]	1.5	8.5	3
	10068172	Anal pruritus	7.9	11.9	7.9	8.0 [2.6, 24.7]	1	2.5	3
	10002958	Aphthous stomatitis	5.3	6.6	5.3	5.3 [1.7, 16.5]	0.8	1.7	3
	10006326	Breath odor	5.2	6.4	5.2	5.2 [1.7, 16.1]	0.8	1.7	3
	10061971	Gastritis bacterial	29.3	55.4	28.9	29.3 [9.4, 91.6]	1.6	9.3	3
	10030997	Oral mucosal eruption	8.5	13.1	8.5	8.5 [2.7, 26.5]	1	2.7	3
	10052894	Oral pruritus	5.6	7.2	5.6	5.6 [1.8, 17.4]	0.8	1.8	3
	10056674	Oral pustule	64.2	125.2	62.4	64.2 [20.4, 202.4]	1.9	19.8	3
	10052453	Palatal disorder	21.5	39.6	21.3	21.5 [6.9, 67.2]	1.4	6.8	3
	10043945	Tongue coated	8.4	12.8	8.4	8.4 [2.7, 26.1]	1	2.7	3
	10070072	Tongue pruritus	16.8	30.0	16.7	16.8 [5.4, 52.4]	1.3	5.4	3
	10047708	Vomiting projectile	4.9	5.7	4.9	4.9 [1.6, 15.1]	0.7	1.6	3
General disorders and administration site conditions	10037660	Pyrexia	3.2	348.2	3.2	3.3 [2.9, 3.7]	1.5	2.8	235
	10016029	Face edema	27.5	2646.5	27.1	28.0 [23.1, 33.9]	4	22.4	106
	10049438	General physical health deterioration	2.5	50.0	2.5	2.5 [1.9, 3.2]	1	1.9	59
	10042682	Swelling face	3.6	95.3	3.6	3.6 [2.7, 4.7]	1.4	2.7	53
	10030095	Edema	4.2	121.3	4.2	4.2 [3.2, 5.6]	1.6	3.2	51
	10061218	Inflammation	2.5	21.6	2.5	2.5 [1.7, 3.7]	0.9	1.7	26
	10059866	Drug resistance	3.6	45.3	3.6	3.7 [2.5, 5.4]	1.3	2.5	25
	10077361	Multiple organ dysfunction syndrome	4.2	54.7	4.2	4.2 [2.8, 6.2]	1.4	2.7	24
	10018092	Generalized edema	7.9	120.2	7.9	8.0 [5.2, 12.2]	1.9	5.2	21
	10028116	Mucosal inflammation	2.3	8.7	2.3	2.4 [1.4, 4.1]	0.7	1.4	13
	10048961	Localized edema	10.5	76.4	10.5	10.5 [5.6, 19.6]	1.8	5.6	10
	10069221	Product color issue	6.5	32.1	6.5	6.5 [3.3, 13.1]	1.4	3.3	8
	10053459	Secretion discharge	3.0	7.3	3.0	3.0 [1.4, 6.2]	0.7	1.4	7
	10020741	Hyperpyrexia	7.1	25.6	7.1	7.1 [3.2, 15.9]	1.3	3.2	6
	10039740	Screaming	3.2	5.4	3.2	3.2 [1.3, 7.7]	0.7	1.3	5
	10014579	Enanthema	33.9	95.5	33.4	33.9 [12.6, 91.1]	1.9	12.4	4
	10049998	Idiosyncratic drug reaction	14.4	37.3	14.4	14.5 [5.4, 38.7]	1.4	5.4	4
	10028133	Mucous membrane disorder	11.3	27.7	11.2	11.3 [4.2, 30.1]	1.3	4.2	4
	10048723	Multiple-drug resistance	4.8	8.6	4.8	4.8 [1.8, 12.9]	0.9	1.8	4
	10030111	Edema mucosal	13.0	22.2	12.9	13.0 [4.2, 40.5]	1.2	4.2	3
Hepatobiliary disorders	10023126	Jaundice	18.6	1967.8	18.5	19.0 [15.8, 22.8]	3.5	15.4	120
	10019754	Hepatitis cholestatic	85.4	8118.0	82.3	87.0 [71.2, 106.2]	5.2	67.4	102
	10008635	Cholestasis	21.0	1507.1	20.8	21.3 [17.1, 26.5]	3.5	16.7	81
	10019717	Hepatitis	11.4	641.8	11.3	11.5 [9.1, 14.6]	2.8	8.9	69
	10024690	Liver function test abnormal	5.2	129.9	5.2	5.2 [3.8, 7.1]	1.7	3.8	40
	10060795	Hepatic enzyme increased	2.6	35.3	2.6	2.6 [1.9, 3.5]	1	1.9	39
	10067125	Liver injury	6.8	178.6	6.8	6.9 [5, 9.5]	2	4.9	37
	10019837	Hepatocellular injury	11.5	303.1	11.4	11.5 [8.2, 16.2]	2.5	8.1	33
	10023129	Jaundice cholestatic	41.2	1156.0	40.5	41.5 [29, 59.2]	3.7	28.4	31
	10020578	Hyperbilirubinemia	13.6	336.0	13.5	13.7 [9.5, 19.6]	2.6	9.4	30
	10003827	Autoimmune hepatitis	16.6	377.4	16.5	16.7 [11.4, 24.4]	2.8	11.3	27
	10019663	Hepatic failure	3.6	48.7	3.6	3.6 [2.5, 5.3]	1.3	2.5	27
	10019727	Hepatitis acute	17.4	382.8	17.3	17.5 [11.9, 25.7]	2.8	11.7	26
	10019670	Hepatic function abnormal	2.7	22.8	2.7	2.7 [1.8, 4.1]	0.9	1.8	23
	10019851	Hepatotoxicity	3.8	42.2	3.8	3.8 [2.5, 5.8]	1.3	2.5	22
	10019708	Hepatic steatosis	4.8	52.8	4.8	4.8 [3, 7.5]	1.4	3	19
	10067969	Cholestatic liver injury	37.7	596.1	37.0	37.8 [23.7, 60.2]	3.3	23.2	18
	10066758	Mixed liver injury	32.3	507.4	31.8	32.4 [20.3, 51.6]	3.1	20	18
	10000804	Acute hepatic failure	4.6	40.9	4.6	4.6 [2.8, 7.5]	1.3	2.8	16
	10049199	Hepatic cytolysis	6.2	60.7	6.2	6.3 [3.8, 10.4]	1.6	3.7	15
	10071634	Deficiency of bile secretion	561.7	4486.5	449.5	562.8 [290.5, 1090.2]	4.5	232	11
	10019795	Hepatitis toxic	17.8	156.7	17.7	17.8 [9.8, 32.3]	2.3	9.8	11
	10019842	Hepatomegaly	4.0	17.0	3.9	4.0 [2.1, 7.6]	1	2.1	9
	10061998	Hepatic lesion	8.4	44.9	8.4	8.4 [4.2, 16.9]	1.5	4.2	8
	10019692	Hepatic necrosis	7.5	33.1	7.5	7.5 [3.6, 15.8]	1.4	3.6	7
	10019668	Hepatic fibrosis	7.4	27.3	7.4	7.5 [3.3, 16.6]	1.3	3.3	6
	10024714	Liver transplant	6.2	21.3	6.2	6.2 [2.8, 13.9]	1.2	2.8	6
	10004685	Bilirubin conjugated increased	9.9	31.6	9.9	9.9 [4.1, 23.9]	1.4	4.1	5
	10008604	Cholangitis	3.9	8.1	3.9	3.9 [1.6, 9.4]	0.8	1.6	5
	10011411	Cross-sensitivity reaction	27.9	102.8	27.5	27.9 [11.5, 67.4]	2	11.4	5
	10008605	Cholangitis acute	27.6	76.6	27.2	27.6 [10.3, 74]	1.8	10.2	4
	10008909	Chronic hepatitis	15.1	26.4	15.0	15.1 [4.8, 47]	1.3	4.8	3
	10050904	Cytolytic hepatitis	4.3	4.7	4.3	4.3 [1.4, 13.4]	0.7	1.4	3
	10056956	Subacute hepatic failure	77.5	151.2	74.9	77.5 [24.5, 245.1]	2	23.7	3
Immune system disorders	10013700	Drug hypersensitivity	11.4	6507.1	11.3	12.8 [11.8, 13.9]	3.2	10.4	683
	10020751	Hypersensitivity	5.6	923.0	5.6	5.8 [5.1, 6.6]	2.2	4.9	245
	10002198	Anaphylactic reaction	12.9	1791.8	12.8	13.3 [11.3, 15.5]	3.2	11	165
	10002199	Anaphylactic shock	21.4	2314.0	21.2	21.9 [18.2, 26.2]	3.7	17.7	121
	10053613	Type IV hypersensitivity reaction	77.8	3811.3	75.3	78.6 [59.7, 103.4]	4.7	57.2	53
	10079645	Therapeutic product cross-reactivity	88.2	1990.3	84.9	88.6 [59.3, 132.2]	4.3	56.9	25
	10045240	Type I hypersensitivity	29.1	642.7	28.8	29.2 [19.7, 43.4]	3.3	19.4	25
	10040402	Serum-sickness-like reaction	81.4	844.2	78.6	81.6 [45.9, 145.3]	3.5	44.2	12
	10047115	Vasculitis	4.4	28.4	4.4	4.4 [2.5, 7.8]	1.2	2.5	12
	10071583	Hemophagocytic lymphohistiocytosis	6.2	34.2	6.2	6.2 [3.2, 12]	1.4	3.2	9
	10002216	Anaphylactoid reaction	8.1	43.0	8.1	8.1 [4.1, 16.3]	1.5	4	8
	10051379	Systemic inflammatory response syndrome	8.9	41.3	8.9	8.9 [4.2, 18.7]	1.5	4.2	7
Infections and infestations	10054236	Clostridium difficile infection	13.9	632.4	13.9	14.1 [10.8, 18.4]	2.9	10.6	54
	10009657	Clostridium difficile colitis	22.8	948.1	22.6	23.0 [17.2, 30.7]	3.4	16.9	47
	10040070	Septic shock	3.8	68.8	3.8	3.9 [2.8, 5.4]	1.4	2.7	34
	10017533	Fungal infection	3.7	54.8	3.7	3.7 [2.6, 5.4]	1.3	2.6	29
	10074170	Candida infection	8.0	152.0	8.0	8.0 [5.5, 11.8]	2	5.4	26
	10034133	Pathogen resistance	9.9	182.8	9.9	10.0 [6.7, 14.9]	2.2	6.6	24
	10027201	Meningitis aseptic	22.3	403.0	22.1	22.4 [14.6, 34.5]	2.9	14.4	21
	10061126	Escherichia infection	8.3	82.5	8.3	8.3 [4.9, 14.1]	1.8	4.9	14
	10061259	Klebsiella infection	14.1	156.2	14.0	14.1 [8.3, 23.9]	2.2	8.3	14
	10037128	Pseudomembranous colitis	27.5	302.0	27.2	27.5 [15.9, 47.6]	2.8	15.7	13
	10064899	Vulvovaginal mycotic infection	12.5	104.5	12.4	12.5 [6.9, 22.6]	2	6.9	11
	10061043	Clostridial infection	8.6	53.1	8.6	8.6 [4.5, 16.6]	1.6	4.5	9
	10015108	Epstein–Barr virus infection	6.6	36.9	6.5	6.6 [3.4, 12.6]	1.4	3.4	9
	10058305	Clostridium colitis	25.7	140.5	25.4	25.7 [12.2, 54.1]	2.2	12	7
	10021914	Infectious mononucleosis	13.9	71.0	13.8	13.9 [6.6, 29.3]	1.8	6.6	7
	10061471	Pseudomonas infection	4.2	13.8	4.2	4.2 [2, 8.8]	1	2	7
	10014568	Empyema	14.1	59.9	14.0	14.1 [6.3, 31.4]	1.7	6.3	6
	10061124	Enterococcal infection	5.4	13.7	5.4	5.4 [2.2, 13]	1	2.2	5
	10034686	Peritonsillar abscess	28.5	105.3	28.2	28.5 [11.8, 69]	2	11.7	5
	10068556	Anal fungal infection	230.4	633.6	209.1	230.6 [82.4, 645.5	2.8	74.7	4
	10053555	Arthritis bacterial	4.3	7.2	4.3	4.3 [1.6, 11.6]	0.8	1.6	4
	10007152	Candidiasis	3.6	5.2	3.6	3.6 [1.4, 9.7]	0.7	1.4	4
	10037569	Purulent discharge	4.8	8.4	4.8	4.8 [1.8, 12.7]	0.8	1.8	4
	10062279	Urinary tract infection pseudomonal	33.9	95.5	33.4	33.9 [12.6, 91.1]	1.9	12.4	4
	10052815	Antibiotic-associated colitis	518.5	873.4	421.4	518.7 [147.8, 1820.8]	2.5	120	3
	10057847	Biliary sepsis	27.7	52.2	27.4	27.8 [8.9, 86.7]	1.5	8.8	3
	10006105	Brain abscess	4.9	5.9	4.9	4.9 [1.6, 15.3]	0.7	1.6	3
	10069657	*Burkholderia cepacia* complex infection	45.8	88.8	45.0	45.9 [14.6, 143.9]	1.7	14.3	3
	10069748	*Burkholderia pseudomallei* infection	224.7	420.9	204.3	224.8 [68.6, 736.8]	2.3	62.3	3
	10058666	Cytomegalovirus infection reactivation	4.5	5.0	4.5	4.5 [1.4, 13.9]	0.7	1.4	3
	10015296	Escherichia sepsis	5.2	6.4	5.2	5.2 [1.7, 16.2]	0.7	1.7	3
	10058017	Infective aneurysm	28.4	53.7	28.1	28.5 [9.1, 88.9]	1.5	9	3
	10033079	Otitis media acute	18.6	33.7	18.5	18.6 [6, 58]	1.4	5.9	3
	10053461	Trichosporon infection	30.4	57.6	30.0	30.4 [9.7, 94.9]	1.6	9.6	3
	10062233	Uterine infection	26.0	48.8	25.7	26.0 [8.3, 81.3]	1.5	8.2	3
Injury, poisoning, and procedural complications	10027091	Medication error	2.7	41.7	2.7	2.7 [2, 3.7]	1	2	41
	10081770	Product prescribing error	2.8	18.5	2.8	2.9 [1.8, 4.6]	0.9	1.8	17
	10076470	Documented hypersensitivity to administered product	45.8	344.2	45.0	45.9 [23.7, 88.9]	2.8	23.2	9
	10076245	Transcription medication error	14.2	72.7	14.1	14.2 [6.8, 29.9]	1.8	6.7	7
	10081579	Wrong product administered	3.6	8.9	3.6	3.6 [1.6, 8.1]	0.8	1.6	6
	10017051	Foreign body trauma	17.6	46.6	17.4	17.6 [6.6, 47]	1.5	6.5	4
	10001605	Alcohol poisoning	4.4	4.9	4.4	4.4 [1.4, 13.8]	0.7	1.4	3
Investigations	10001551	Alanine aminotransferase increased	3.6	92.8	3.6	3.7 [2.8, 4.8]	1.4	2.7	50
	10003481	Aspartate aminotransferase increased	3.8	91.8	3.8	3.9 [2.9, 5.2]	1.4	2.9	45
	10024378	Leukocytosis	9.8	307.7	9.8	9.9 [7.2, 13.5]	2.4	7.2	40
	10005364	Blood bilirubin increased	6.1	156.3	6.1	6.1 [4.4, 8.4]	1.9	4.4	38
	10059570	Blood alkaline phosphatase increased	6.5	162.9	6.5	6.6 [4.7, 9.1]	1.9	4.7	36
	10006825	C-reactive protein increased	5.0	105.8	5.0	5.1 [3.6, 7.1]	1.7	3.6	34
	10017693	Gamma-glutamyl transferase increased	7.2	169.3	7.2	7.2 [5.1, 10.2]	2	5.1	33
	10022595	International normalized ratio increased	4.3	75.4	4.3	4.3 [3, 6.2]	1.5	3	31
	10014950	Eosinophilia	7.2	136.9	7.1	7.2 [4.9, 10.5]	1.9	4.9	27
	10024384	Leukopenia	2.4	19.7	2.4	2.4 [1.6, 3.5]	0.9	1.6	26
	10054889	Transaminases increased	4.6	63.9	4.6	4.6 [3.1, 6.9]	1.5	3.1	24
	10021015	Hypokalemia	2.1	10.4	2.1	2.1 [1.4, 3.3]	0.7	1.3	20
	10001507	Agranulocytosis	4.9	55.5	4.9	4.9 [3.1, 7.8]	1.5	3.1	19
	10005911	Body temperature increased	4.3	44.0	4.2	4.3 [2.7, 6.7]	1.3	2.7	19
	10068237	Hypertransaminasemia	14.2	219.3	14.1	14.3 [9.1, 22.4]	2.4	9	19
	10077692	Liver function test increased	2.3	6.7	2.3	2.3 [1.3, 4.1]	0.7	1.2	11
	10009802	Coagulopathy	2.6	8.0	2.6	2.6 [1.4, 4.8]	0.7	1.4	10
	10021113	Hypothermia	3.6	14.1	3.6	3.6 [1.9, 6.9]	1	1.8	9
	10029379	Neutrophilia	5.6	29.5	5.6	5.6 [2.9, 10.8]	1.3	2.9	9
	10070027	Clostridium test positive	17.8	109.7	17.7	17.8 [8.9, 35.7]	2.1	8.8	8
	10014392	Electrocardiogram ST segment elevation	8.1	36.9	8.1	8.1 [3.9, 17.1]	1.4	3.9	7
	10069826	Inflammatory marker increased	6.9	24.8	6.9	7.0 [3.1, 15.5]	1.3	3.1	6
	10005586	Blood immunoglobulin a increased	36.8	104.2	36.3	36.9 [13.7, 99]	1.9	13.5	4
	10009736	Coagulation factor decreased	59.9	171.9	58.4	60.0 [22.2, 161.9]	2.2	21.6	4
	10062685	Hepatic enzyme abnormal	3.3	4.2	3.3	3.3 [1.2, 8.7]	0.6	1.2	4
	10050791	Leukocyturia	24.4	67.2	24.2	24.4 [9.1, 65.5]	1.7	9	4
	10063342	Tryptase increased	26.4	73.0	26.1	26.4 [9.8, 70.7]	1.8	9.7	4
	10009799	Coagulation time prolonged	7.6	11.1	7.5	7.6 [2.4, 23.5]	0.9	2.4	3
	10067081	Procalcitonin increased	13.4	23.0	13.3	13.4 [4.3, 41.7]	1.2	4.3	3
	10060888	Skin test negative	187.2	355.4	172.9	187.3 [57.7, 608.4]	2.3	53.2	3
	10070055	Streptococcus test positive	13.7	23.7	13.7	13.7 [4.4, 42.7]	1.2	4.4	3
Metabolism and nutrition disorders	10000159	Abnormal loss of weight	3.4	4.5	3.4	3.4 [1.3, 9]	0.7	1.3	4
	10057248	Cell death	7.2	10.4	7.2	7.2 [2.3, 22.4]	0.9	2.3	3
	10021083	Hypoproteinemia	9.1	14.2	9.1	9.1 [2.9, 28.3]	1	2.9	3
Musculoskeletal and connective tissue disorders	10003267	Arthritis reactive	20.1	36.7	19.9	20.1 [6.5, 62.7]	1.4	6.4	3
Nervous system disorders	10036653	Presyncope	3.2	23.4	3.2	3.2 [2, 5.1]	1	2	17
	10040108	Serotonin syndrome	2.5	10.7	2.5	2.6 [1.5, 4.4]	0.8	1.5	13
	10027199	Meningitis	3.1	5.2	3.1	3.1 [1.3, 7.5]	0.7	1.3	5
	10042264	Stupor	4.7	8.3	4.7	4.7 [1.8, 12.7]	0.8	1.8	4
	10003805	Autism	8.4	12.8	8.4	8.4 [2.7, 26.1]	1	2.7	3
	10070511	Hypoxic–ischemic encephalopathy	4.7	5.4	4.7	4.7 [1.5, 14.6]	0.7	1.5	3
	10074869	Language disorder	5.5	7.0	5.5	5.5 [1.8, 17.1]	1.5	9	3
	10068357	Neurological decompensation	6.1	8.2	6.1	6.1 [2, 19]	0.8	2	3
Pregnancy, puerperium, and perinatal conditions	10036590	Premature baby	2.7	24.4	2.7	2.7 [1.8, 4]	1	1.8	25
	10036595	Premature delivery	2.7	11.3	2.7	2.7 [1.5, 4.8]	0.8	1.5	12
	10049550	Live birth	4.1	13.2	4.1	4.1 [1.9, 8.5]	1	1.9	7
	10030289	Oligohydramnios	4.4	7.3	4.4	4.4 [1.6, 11.7]	0.8	1.6	4
	10073024	Preterm premature rupture of membranes	9.6	15.2	9.6	9.6 [3.1, 29.9]	1	3.1	3
Psychiatric disorders	10026749	Mania	2.2	5.4	2.2	2.2 [1.2, 4.1]	0.6	1.2	10
Renal and urinary disorders	10008796	Chromaturia	11.1	511.1	11.0	11.2 [8.6, 14.5]	2.7	8.5	57
	10048302	Tubulointerstitial nephritis	6.5	141.9	6.4	6.5 [4.6, 9.2]	1.9	4.5	32
	10024515	Linear IgA disease	50.7	1047.0	49.6	50.9 [33.6, 77]	3.7	32.8	23
	10038436	Renal failure acute	2.1	11.3	2.1	2.1 [1.4, 3.3]	0.7	1.4	21
	10008684	Choluria	268.5	4286.7	239.9	269.4 [167.3, 433.8]	4.9	149	19
	10018867	Hematuria	2.4	13.9	2.4	2.4 [1.5, 3.7]	0.8	1.5	19
	10013990	Dysuria	2.2	10.0	2.2	2.2 [1.4, 3.4]	0.7	1.4	18
	10002847	Anuria	3.0	6.0	3.0	3.0 [1.3, 6.6]	0.7	1.3	6
	10030302	Oliguria	4.3	12.0	4.3	4.3 [1.9, 9.6]	0.9	1.9	6
	10071503	Crystal nephropathy	25.0	68.8	24.7	25.0 [9.3, 66.9]	1.7	9.2	4
Reproductive system and breast disorders	10037093	Pruritus genital	20.0	143.0	19.9	20.1 [10.4, 38.7]	2.2	10.3	9
	10054816	Genital erythema	76.8	507.8	74.3	76.9 [38, 155.7]	3.1	36.7	8
	10047784	Vulvovaginal candidiasis	22.7	102.6	22.5	22.7 [10.2, 50.8]	2	10.1	6
	10018175	Genital rash	11.9	29.6	11.8	11.9 [4.5, 31.8]	1.3	4.4	4
Reproductive system and breast disorders	10047768	Vulval ulceration	57.2	164.2	55.8	57.3 [21.2, 154.6]	2.2	20.7	4
	10004078	Balanoposthitis	16.3	28.9	16.2	16.3 [5.2, 50.7]	1.3	5.2	3
	10030104	Edema genital	19.3	35.0	19.1	19.3 [6.2, 60]	1.4	6.1	3
Respiratory, thoracic, and mediastinal disorders	10013968	Dyspnea	2.5	279.5	2.5	2.5 [2.3, 2.9]	1.2	2.2	317
	10006482	Bronchospasm	12.2	366.2	12.1	12.2 [8.8, 16.9]	2.6	8.8	37
	10023845	Laryngeal oedema	20.5	513.2	20.3	20.6 [14.3, 29.7]	3	14.1	29
	10047924	Wheezing	2.4	22.2	2.4	2.4 [1.7, 3.5]	0.9	1.7	29
	10013952	Dysphonia	2.1	14.8	2.1	2.1 [1.5, 3.1]	0.7	1.4	27
	10021143	Hypoxia	2.7	19.6	2.7	2.7 [1.7, 4.2]	0.9	1.7	20
	10011703	Cyanosis	5.2	60.4	5.2	5.2 [3.3, 8.2]	1.5	3.3	19
	10038687	Respiratory distress	2.2	8.4	2.2	2.2 [1.3, 3.8]	0.7	1.3	14
	10043089	Tachypnea	4.2	28.3	4.2	4.2 [2.4, 7.2]	1.2	2.4	13
	10084380	COVID-19 pneumonia	2.5	5.0	2.5	2.5 [1.2, 5.3]	0.6	1.2	7
	10080148	Eosinophilic pleural effusion	85.9	406.4	82.7	85.9 [38, 194.3]	2.8	36.6	6
	10006473	Bronchopulmonary aspergillosis	3.0	5.0	3.0	3.0 [1.3, 7.3]	0.7	1.3	5
	10008590	Choking sensation	4.1	8.8	4.1	4.1 [1.7, 9.9]	0.8	1.7	5
	10006469	Bronchopneumonia	4.9	8.7	4.9	4.9 [1.8, 13]	0.9	1.8	4
	10006487	Bronchostenosis	35.9	101.6	35.4	36.0 [13.4, 96.6]	1.9	13.2	4
	10035724	Pneumonia mycoplasmal	25.9	71.6	25.6	25.9 [9.7, 69.5]	1.7	9.6	4
	10035728	Pneumonia pneumococcal	13.2	33.4	13.1	13.2 [4.9, 35.2]	1.4	4.9	4
	10037833	Rales	3.6	5.0	3.6	3.6 [1.3, 9.5]	0.7	1.3	4
	10044003	Tonsillar hypertrophy	10.1	24.2	10.1	10.1 [3.8, 27]	1.2	3.8	4
	10064779	Breath sounds	114.2	221.7	108.8	114.3 [35.8, 364.7]	2.1	34.1	3
	10006440	Bronchial obstruction	10.5	17.0	10.4	10.5 [3.4, 32.6]	1.1	3.4	3
	10023891	Laryngospasm	5.0	6.0	5.0	5.0 [1.6, 15.5]	0.7	1.6	3
	10081792	Lung opacity	6.3	8.5	6.2	6.3 [2, 19.5]	0.9	2	3
	10078257	Tonsillar exudate	374.4	664.2	321.1	374.6 [110.3, 1272.2]	2.5	94.6	3
Skin and subcutaneous tissue disorders	10037087	Pruritus	5.2	1456.7	5.2	5.6 [5, 6.2]	2.2	4.7	422
	10037844	Rash	3.5	649.6	3.5	3.7 [3.3, 4.1]	1.6	3.2	354
	10046735	Urticaria	8.2	1985.7	8.2	8.6 [7.7, 9.7]	2.7	7.3	314
	10015150	Erythema	6.0	903.9	6.0	6.2 [5.4, 7.1]	2.3	5.2	218
	10037868	Rash maculo-papular	39.2	6737.3	38.5	40.5 [34.9, 47]	4.5	33.3	185
	10073508	Drug reaction with eosinophilia and systemic symptoms	17.5	1778.2	17.4	17.9 [14.9, 21.5]	3.4	14.5	116
	10037884	Rash pruritic	8.7	687.2	8.7	8.9 [7.3, 10.8]	2.6	7.1	102
	10002424	Angioedema	8.2	627.2	8.2	8.4 [6.9, 10.2]	2.5	6.7	100
	10037858	Rash generalized	8.2	345.9	8.2	8.3 [6.4, 10.8]	2.3	6.3	56
	10048799	Acute generalized exanthematous pustulosis	27.9	1333.7	27.6	28.2 [21.5, 37]	3.6	21	53
	10037855	Rash erythematous	5.5	184.6	5.5	5.6 [4.2, 7.3]	1.9	4.2	51
	10052576	Pruritus generalized	8.9	326.7	8.8	8.9 [6.7, 11.9]	2.4	6.7	48
	10040882	Skin lesion	6.7	199.6	6.7	6.8 [5, 9.2]	2	5	42
	10044223	Toxic epidermal necrolysis	11.6	392.6	11.5	11.6 [8.6, 15.8]	2.6	8.5	42
	10040844	Skin exfoliation	3.0	51.3	3.0	3.0 [2.2, 4.1]	1.2	2.2	40
	10005191	Blister	3.3	59.6	3.3	3.3 [2.4, 4.5]	1.2	2.4	39
	10013687	Drug eruption	9.5	263.0	9.4	9.5 [6.9, 13.2]	2.3	6.8	36
	10057970	Toxic skin eruption	18.3	566.1	18.1	18.4 [13.2, 25.5]	3	13	36
	10037867	Rash macular	4.8	92.1	4.8	4.8 [3.4, 6.8]	1.6	3.4	32
	10040914	Skin reaction	9.5	225.3	9.4	9.5 [6.7, 13.5]	2.3	6.6	31
	10042033	Stevens–Johnson syndrome	5.0	95.1	5.0	5.0 [3.5, 7.2]	1.6	3.5	31
	10037888	Rash pustular	18.6	477.4	18.4	18.7 [13, 26.8]	2.9	12.8	30
	10015218	Erythema multiforme	11.4	235.2	11.3	11.4 [7.8, 16.8]	2.4	7.7	26
	10012434	Dermatitis allergic	7.9	126.4	7.9	8.0 [5.2, 12.1]	2	5.2	22
	10037876	Rash papular	5.7	80.2	5.7	5.7 [3.8, 8.7]	1.6	3.7	22
	10012441	Dermatitis bullous	11.8	185.9	11.7	11.8 [7.6, 18.4]	2.3	7.6	20
	10037549	Purpura	10.2	148.0	10.2	10.2 [6.5, 16.1]	2.1	6.5	19
	10012455	Dermatitis exfoliative	13.5	171.7	13.4	13.5 [8.3, 22.1]	2.3	8.2	16
	10051576	Generalized erythema	11.4	131.6	11.4	11.4 [6.9, 19]	2.1	6.8	15
	10002473	Angioneurotic edema	16.9	191.8	16.7	16.9 [10, 28.6]	2.4	9.9	14
	10012456	Dermatitis exfoliative generalized	19.1	219.9	18.9	19.1 [11.3, 32.3]	2.5	11.2	14
	10078325	Symmetrical drug-related intertriginous and flexural exanthema	70.2	860.3	68.1	70.4 [41.3, 119.9]	3.6	40	14
	10082980	Mucocutaneous disorder	227.2	1640.8	206.4	227.5 [114.6, 451.9]	3.9	104	9
	10034754	Petechiae	4.0	17.0	4.0	4.0 [2.1, 7.6]	1	2.1	9
	10037578	Pustule	17.6	108.6	17.5	17.7 [8.8, 35.4]	2.1	8.7	8
	10016741	Fixed eruption	16.3	85.2	16.2	16.3 [7.8, 34.3]	1.9	7.7	7
	10054107	Nodule	2.5	4.7	2.5	2.5 [1.2, 5.2]	0.6	1.2	7
	10034277	Pemphigoid	4.4	14.9	4.4	4.4 [2.1, 9.2]	1	2.1	7
	10037870	Rash morbilliform	11.1	54.5	11.1	11.1 [5.3, 23.4]	1.7	5.3	7
	10058679	Skin edema	32.0	177.6	31.5	32.0 [15.2, 67.5]	2.4	14.9	7
	10048245	Yellow skin	6.4	26.6	6.4	6.4 [3, 13.4]	1.3	3	7
	10011686	Cutaneous vasculitis	7.6	28.0	7.6	7.6 [3.4, 17]	1.3	3.4	6
	10067868	Skin mass	4.2	11.6	4.2	4.2 [1.9, 9.4]	0.9	1.9	6
	10014141	Ecthyma	50.6	191.7	49.5	50.6 [20.9, 122.9]	2.3	20.4	5
	10015226	Erythema nodosum	6.2	17.0	6.2	6.2 [2.6, 15]	1.1	2.6	5
	10084905	Generalized bullous fixed drug eruption	70.2	267.0	68.1	70.3 [28.8, 171.2]	2.5	28	5
	10020764	Hypersensitivity vasculitis	7.5	21.9	7.5	7.5 [3.1, 18]	1.2	3.1	5
	10037083	Prurigo	30.0	111.3	29.7	30.1 [12.4, 72.7]	2	12.3	5
	10040893	Skin necrosis	4.1	8.8	4.1	4.1 [1.7, 9.8]	0.8	1.7	5
	10042343	Subcutaneous abscess	4.8	11.4	4.8	4.8 [2, 11.5]	0.9	2	5
	10047642	Vitiligo	9.4	29.3	9.3	9.4 [3.9, 22.5]	1.3	3.9	5
	10037898	Rash vesicular	3.9	6.0	3.9	3.9 [1.5, 10.4]	0.7	1.5	4
	10053262	Skin swelling	3.3	4.4	3.3	3.3 [1.2, 8.9]	0.6	1.2	4
	10047097	Vascular purpura	14.7	38.1	14.6	14.7 [5.5, 39.4]	1.4	5.5	4
	10047476	Viral rash	46.8	133.6	45.9	46.8 [17.4, 126.1]	2	17	4
	10000748	Acute febrile neutrophilic dermatosis	5.0	6.0	5.0	5.0 [1.6, 15.5]	0.7	1.6	3
	10070599	Acute hemorrhagic edema of infancy	337.0	606.0	293.2	337.2 [100.2, 1135]	2.4	87	3
	10009869	Cold urticaria	55.7	108.4	54.4	55.7 [17.7, 175.3]	1.8	17.3	3
	10019617	Henoch–Schönlein purpura	6.2	8.4	6.2	6.2 [2, 19.4]	0.8	2	3
	10064000	Lichenoid keratosis	6.1	8.2	6.1	6.1 [2, 19]	0.8	2	3
	10029415	Nikolsky’s sign	25.1	46.8	24.8	25.1 [8, 78.2]	1.4	7	3
	10035039	Piloerection	11.9	20.0	11.9	11.9 [3.8, 37.1]	1.1	3.8	3
	10046742	Urticaria contact	396.5	697.7	337.1	396.7 [116.2, 1354]	2.5	98.8	3
	10052569	Urticaria generalized	14.9	26.1	14.8	14.9 [4.8, 46.4]	1.2	4.8	3
Surgical and medical procedures	10050729	Self-medication	13.8	176.9	13.8	13.9 [8.5, 22.7]	2.3	8.4	16
Vascular disorders	10021097	Hypotension	2.2	65.1	2.2	2.2 [1.8, 2.7]	0.9	1.8	101
	10009192	Circulatory collapse	3.6	26.1	3.6	3.6 [2.2, 6.1]	1.1	2.2	15
	10018852	Hematoma	2.5	12.5	2.5	2.5 [1.5, 4.2]	0.8	1.5	15
	10033546	Pallor	2.3	9.7	2.3	2.3 [1.4, 3.8]	0.7	1.4	15
	10020565	Hyperemia	17.6	108.3	17.5	17.6 [8.8, 35.3]	2.1	8.7	8
	10052076	Hemodynamic instability	2.8	4.0	2.8	2.8 [1.2, 6.7]	0.6	1.2	5
	10034636	Peripheral vascular disorder	3.2	4.1	3.2	3.2 [1.2, 8.6]	0.6	1.2	4
	10034567	Peripheral circulatory failure	37.4	71.9	36.8	37.5 [12, 117.3]	1.7	11.8	3
	10048820	Urticarial vasculitis	26.4	49.6	26.1	26.4 [8.5, 82.6]	1.5	8.4	3

SOC: System organ classification; MedDRA: Medical dictionary of adverse events; PRR: Proportional reporting ratio; χ^2^: Chi-square test; RRR: Relative reporting ratio; ROR: Reporting odds ratio; IC: Information component; and EBGM: Empirical Bayes Geometric Mean.

**Table 5:** Signal detection measures for adverse events reported with relebactam.

SOC	MedDRA	Adverse Event	PRR	χ^2^	RRR	ROR [95% CI]	IC025	EBGM05	Number of Cases
General disorders and administration site conditions	10011906	Death	4.0	15.7	4.0	4.7 [2.2, 10.1]	0.9	1.8	8
Injury, poisoning, and procedural complications	10076476	Product use in unapproved indication	11.1	36.6	11.1	12.4 [4.9, 31.5]	1.4	4.4	5
	10053762	Off-label use	8.3	79.4	8.3	11.5 [6, 22]	1.6	4.3	13
Infections and infestations	10034133	Pathogen resistance	161.5	331.4	161.4	173.5 [53.7, 561]	2.3	49.9	3
Nervous system disorder	10015037	Epilepsy	71.3	211.5	71.2	78.5 [28, 219.6]	2.2	25.5	4

SOC: System organ classification; MedDRA: Medical dictionary of adverse events; PRR: Proportional reporting ratio; χ^2^: Chi-square test; RRR: Relative reporting ratio; ROR: Reporting odds ratio; IC: Information component; and EBGM: Empirical Bayes Geometric Mean.

**Table 6:** Signal detection measures for adverse events reported with sulbactam.

SOC	MedDRA Code	Adverse Event	PRR	χ^2^	RRR	ROR [95% CI]	IC025	EBHM05	Number of Cases
Blood and lymphatic system disorders	10018916	Hemolytic anemia	19.5	87.4	19.4	19.6 [8.8, 43.8]	1.9	8.7	6
	10025197	Lymphadenopathy	4.3	9.7	4.3	4.3 [1.8, 10.5]	0.9	1.8	5
	10013442	Disseminated intravascular coagulation	8.6	19.9	8.6	8.7 [3.2, 23.2]	1.2	3.2	4
	10062713	Hemorrhagic diathesis	25.1	47.3	25.1	25.2 [8.1, 78.4]	1.5	8.1	3
Cardiac disorders	10007515	Cardiac arrest	3.7	19.2	3.7	3.7 [2.1, 6.8]	1	2	11
	10043071	Tachycardia	3.0	10.0	3.0	3.0 [1.6, 5.8]	0.8	1.5	9
	10007617	Cardio-respiratory arrest	5.3	23.7	5.3	5.3 [2.7, 10.7]	1.2	2.6	8
	10069167	Kounis syndrome	95.1	649.5	94.5	96.0 [47.7, 193]	3.3	47	8
	10006093	Bradycardia	3.1	6.4	3.1	3.1 [1.4, 6.9]	0.7	1.4	6
	10047290	Ventricular fibrillation	10.2	24.7	10.2	10.3 [3.8, 27.4]	1.3	3.8	4
	10007625	Cardiogenic shock	6.1	8.2	6.1	6.1 [2, 19]	0.8	2	3
Gastrointestinal disorders	10014896	Enterocolitis hemorrhagic	720.6	25,373.2	687.6	755.4 [541.5, 1053.8]	6.8	492.9	38
	10018836	Hematochezia	3.5	8.3	3.5	3.5 [1.6, 7.8]	0.8	1.6	6
	10024570	Lip swelling	3.4	4.5	3.4	3.4 [1.3, 9]	0.7	1.3	4
	10042727	Swollen tongue	3.4	4.7	3.4	3.5 [1.3, 9.2]	0.7	1.3	4
	10012743	Diarrhea neonatal	493.3	990.0	477.6	495.1 [156.4, 1566.9]	2.8	150.9	3
General disorders and administration site conditions	10037660	Pyrexia	2.2	14.7	2.2	2.2 [1.5, 3.4]	0.8	1.5	24
	10010264	Condition aggravated	2.0	9.2	2.0	2.0 [1.3, 3.2]	0.6	1.3	20
	10016029	Face edema	12.2	61.3	12.2	12.3 [5.9, 25.9]	1.7	5.8	7
	10059866	Drug resistance	6.0	20.5	6.0	6.1 [2.7, 13.6]	1.2	2.7	6
	10020741	Hyperpyrexia	41.3	157.9	41.2	41.5 [17.2, 100.2]	2.2	17.1	5
	10061819	Disease recurrence	3.2	4.1	3.2	3.2 [1.2, 8.6]	0.6	1.2	4
	10028154	Multi-organ failure	5.8	11.4	5.8	5.8 [2.2, 15.5]	0.9	2.2	4
Hepatobiliary disorders	10023126	Jaundice	9.4	59.2	9.4	9.5 [4.9, 18.3]	1.7	4.9	9
	10019670	Hepatic function abnormal	6.4	31.3	6.4	6.5 [3.2, 13]	1.3	3.2	8
	10008635	Cholestasis	12.4	62.3	12.4	12.5 [5.9, 26.3]	1.7	5.9	7
	10019663	Hepatic failure	6.4	26.6	6.4	6.4 [3.1, 13.5]	1.3	3	7
	10072268	Drug-induced liver injury	5.9	19.7	5.9	5.9 [2.7, 13.2]	1.1	2.6	6
	10024670	Liver disorder	3.8	9.6	3.8	3.8 [1.7, 8.5]	0.9	1.7	6
	10024690	Liver function test abnormal	5.2	16.4	5.2	5.2 [2.3, 11.7]	1.1	2.3	6
	10019795	Hepatitis toxic	55.1	213.6	54.9	55.5 [23, 133.8]	2.4	22.8	5
	10023129	Jaundice cholestatic	44.3	170.1	44.2	44.6 [18.5, 107.5]	2.3	18.3	5
	10067125	Liver injury	6.4	17.6	6.4	6.4 [2.7, 15.4]	1.1	2.6	5
	10008612	Cholecystitis	9.9	23.6	9.9	9.9 [3.7, 26.5]	1.2	3.7	4
	10019772	Hepatitis fulminant	40.8	117.6	40.7	41.0 [15.3, 109.6]	2	15.2	4
Immune system disorders	10013700	Drug hypersensitivity	4.9	136.0	4.9	5.2 [3.8, 7]	1.7	3.6	44
	10002198	Anaphylactic reaction	16.6	439.6	16.6	17.2 [12, 24.6]	2.8	11.6	31
	10002199	Anaphylactic shock	36.3	993.6	36.2	37.6 [26.1, 54.2]	3.6	25.1	30
	10020751	Hypersensitivity	2.6	15.8	2.6	2.7 [1.7, 4.3]	0.9	1.6	17
	10002216	Anaphylactoid reaction	20.8	38.4	20.7	20.8 [6.7, 64.8]	1.4	6.7	3
	10053613	Type IV hypersensitivity reaction	29.3	56.0	29.2	29.4 [9.4, 91.3]	1.6	9.4	3
Infections and infestation	10035664	Pneumonia	3.3	50.7	3.3	3.4 [2.4, 4.8]	1.2	2.3	33
	10040047	Sepsis	3.4	17.9	3.4	3.4 [1.9, 6]	1	1.9	12
	10009657	Clostridium difficile colitis	36.4	343.6	36.3	36.9 [20.3, 66.9]	2.9	20	11
	10034133	Pathogen resistance	22.4	142.7	22.4	22.6 [11.3, 45.4]	2.2	11.1	8
	10040070	Septic shock	5.4	21.1	5.4	5.5 [2.6, 11.5]	1.2	2.6	7
	10061043	Clostridial infection	38.4	182.4	38.3	38.7 [17.3, 86.5]	2.4	17.1	6
	10061259	Klebsiella infection	41.5	198.0	41.4	41.8 [18.7, 93.5]	2.4	18.5	6
	10061126	Escherichia infection	16.2	42.9	16.2	16.3 [6.1, 43.5]	1.5	6.1	4
	10037128	Pseudomembranous colitis	56.7	166.2	56.5	57.0 [21.3, 152.5]	2.2	21.1	4
	10058080	Staphylococcal infection	3.7	5.5	3.7	3.7 [1.4, 10]	0.7	1.4	4
	10003997	Bacteremia	8.5	13.0	8.5	8.5 [2.7, 26.5]	1	2.7	3
	10058852	Clostridium bacteremia	444.4	894.3	431.6	446.1 [141.2, 1409.2]	2.8	136.6	3
	10058305	Clostridium colitis	73.0	146.4	72.6	73.3 [23.5, 228.2]	2	23.3	3
	10054236	Clostridium difficile infection	5.4	6.8	5.4	5.4 [1.7, 16.8]	0.8	1.7	3
Injury, poisoning, and procedural complications	10049193	Drug exposure during pregnancy	3.3	7.4	3.3	3.3 [1.5, 7.3]	0.8	1.5	6
	10061355	Poisoning	6.8	9.6	6.8	6.8 [2.2, 21.2]	0.9	2.2	3
Investigations	10011631	Culture stool positive	1039.1	8751.1	971.7	1051.8 [552.1, 2004]	5.2	510	10
	10070091	Klebsiella test positive	582.2	5039.4	560.4	589.3 [312.2, 1112.6]	4.8	297	10
	10043554	Thrombocytopenia	2.8	9.6	2.8	2.8 [1.5, 5.2]	0.8	1.5	10
	10001551	Alanine aminotransferase increased	3.9	14.8	3.9	4.0 [2, 8]	1	2	8
	10003481	Aspartate aminotransferase increased	4.6	19.4	4.6	4.7 [2.3, 9.4]	1.1	2.3	8
	10005734	Blood pressure decreased	3.6	12.7	3.6	3.7 [1.8, 7.3]	0.9	1.8	8
	10070027	Clostridium test positive	129.3	886.4	128.2	130.5 [64.9, 262.7]	3.5	63.7	8
	10005470	Blood creatine phosphokinase increased	6.1	25.1	6.1	6.2 [2.9, 13]	1.2	2.9	7
	10037063	Prothrombin time prolonged	32.8	184.5	32.7	33.0 [15.7, 69.5]	2.4	15.5	7
	10021015	Hypokalemia	4.3	12.1	4.3	4.3 [1.9, 9.6]	0.9	1.9	6
	10033318	Oxygen saturation decreased	3.9	10.3	3.9	3.9 [1.8, 8.8]	0.9	1.8	6
	10005364	Blood bilirubin increased	5.4	13.9	5.4	5.4 [2.3, 13.1]	1	2.2	5
	10009802	Coagulopathy	8.7	26.7	8.7	8.7 [3.6, 21]	1.3	3.6	5
	10020679	Hypernatremia	33.3	125.6	33.2	33.5 [13.9, 80.7]	2.1	13.8	5
	10024378	Leukocytosis	8.3	25.4	8.3	8.4 [3.5, 20.2]	1.3	3.5	5
	10033661	Pancytopenia	2.8	4.2	2.8	2.8 [1.2, 6.8]	0.6	1.2	5
	10047943	White blood cell count increased	4.1	8.7	4.1	4.1 [1.7, 9.8]	0.8	1.7	5
	10000636	Activated partial thromboplastin time prolonged	22.4	61.7	22.4	22.5 [8.4, 60.1]	1.7	8.4	4
	10005851	Blood urea increased	7.8	17.3	7.7	7.8 [2.9, 20.8]	1.1	2.9	4
	10006825	C-reactive protein increased	4.0	6.4	4.0	4.1 [1.5, 10.9]	0.8	1.5	4
	10014950	Eosinophilia	7.3	15.9	7.3	7.3 [2.7, 19.6]	1.1	2.7	4
	10005630	Blood lactate dehydrogenase increased	6.3	8.5	6.3	6.3 [2, 19.5]	0.9	2	3
	10005728	Blood pressure abnormal	4.4	4.9	4.4	4.5 [1.4, 13.9]	0.7	1.4	3
	10005758	Blood pressure systolic decreased	20.0	36.7	20.0	20.0 [6.4, 62.3]	1.4	6.4	3
	10005911	Body temperature increased	4.6	5.1	4.6	4.6 [1.5, 14.2]	0.7	1.5	3
	10018838	Hematocrit decreased	4.7	5.4	4.7	4.7 [1.5, 14.7]	0.7	1.5	3
Metabolism and nutrition disorders	10001598	Alcohol intolerance	309.3	2116.2	303.1	312.3 [154.6, 631.1]	4.1	150	8
Musculoskeletal and connective tissue disorders	10039020	Rhabdomyolysis	3.2	5.5	3.2	3.2 [1.3, 7.7]	0.7	1.3	5
Nervous system disorders	10010904	Convulsion	3.1	9.1	3.1	3.1 [1.5, 6.2]	0.8	1.5	8
	10001854	Altered state of consciousness	4.2	4.4	4.2	4.2 [1.3, 13]	0.7	1.3	3
	10048962	Brain edema	6.9	9.8	6.9	6.9 [2.2, 21.5]	0.9	2.2	3
Renal and urinary disorders	10062237	Renal impairment	5.6	60.2	5.6	5.7 [3.5, 9.2]	1.5	3.5	17
	10038436	Renal failure acute	7.4	54.5	7.4	7.5 [4.1, 13.5]	1.6	4.1	11
	10018867	Hematuria	3.4	4.6	3.4	3.4 [1.3, 9.1]	0.7	1.3	4
	10038540	Renal tubular necrosis	9.7	15.6	9.7	9.8 [3.1, 30.4]	1.1	3.1	3
Respiratory, thoracic, and mediastinal disorders	10022611	Interstitial lung disease	9.6	106.8	9.6	9.7 [5.8, 16.2]	2	5.7	15
	10001053	Acute respiratory failure	14.1	84.6	14.1	14.2 [7.1, 28.5]	1.9	7	8
	10006482	Bronchospasm	17.8	110.8	17.8	18.0 [9, 36.1]	2.1	8.9	8
	10035598	Pleural effusion	3.1	6.4	3.1	3.1 [1.4, 6.9]	0.7	1.4	6
	10021143	Hypoxia	3.7	5.4	3.7	3.7 [1.4, 9.9]	0.7	1.4	4
	10023845	Laryngeal edema	19.2	51.9	19.2	19.3 [7.2, 51.5]	1.6	7.2	4
	10023891	Laryngospasm	45.9	133.1	45.7	46.1 [17.2, 123.3]	2.1	17.1	4
	10038678	Respiratory depression	8.0	18.0	8.0	8.0 [3. 21.4]	1.1	3	4
	10043089	Tachypnea	6.6	9.3	6.6	6.6 [2.1, 20.6]	0.9	2.1	3
Skin and subcutaneous tissue disorders	10037844	Rash	2.9	50.1	2.9	3.0 [2.2, 4]	1.1	2.1	42
	10037087	Pruritus	3.1	49.2	3.1	3.2 [2.3, 4.4]	1.2	2.2	36
	10015150	Erythema	3.8	38.0	3.8	3.8 [2.5, 6]	1.2	2.4	20
	10037855	Rash erythematous	12.8	173.8	12.8	13.1 [8.1, 21.1]	2.3	7.9	17
	10013687	Drug eruption	26.9	348.0	26.8	27.4 [16.4, 45.6]	2.8	16.1	15
	10044223	Toxic epidermal necrolysis	28.0	363.8	28.0	28.5 [17.1, 47.5]	2.9	16.8	15
	10042033	Stevens–Johnson syndrome	15.2	172.3	15.2	15.5 [9.1, 26.3]	2.3	9	14
	10046735	Urticaria	2.5	11.1	2.5	2.5 [1.5, 4.3]	0.8	1.5	14
	10037868	Rash maculo-papular	18.6	199.5	18.6	18.9 [10.9, 32.7]	2.4	10.8	13
	10037858	Rash generalized	10.7	87.3	10.7	10.8 [6, 19.6]	1.9	5.9	11
	10002424	Angioedema	5.1	25.4	5.0	5.1 [2.6, 9.8]	1.2	2.6	9
	10073508	Drug reaction with eosinophilia and systemic symptoms	8.3	44.0	8.2	8.3 [4.1, 16.7]	1.5	4.1	8
	10048799	Acute generalized exanthematous pustulosis	25.0	137.8	24.9	25.2 [12. 53]	2.2	11.8	7
	10051576	Generalized erythema	30.3	141.7	30.2	30.5 [13.7, 68.2]	2.2	13.5	6
	10024515	Linear IgA disease	88.4	432.0	87.9	89.0 [39.8, 199.2]	2.9	39.3	6
	10037867	Rash macular	6.2	21.2	6.2	6.2 [2.8, 13.9]	1.2	2.8	6
	10037549	Purpura	14.6	37.8	14.5	14.6 [5.5, 39.1]	1.4	5.4	4
	10057970	Toxic skin eruption	13.9	35.7	13.8	13.9 [5.2, 37.2]	1.4	5.2	4
	10012434	Dermatitis allergic	7.4	10.9	7.4	7.5 [2.4, 23.3]	0.9	2.4	3
	10033733	Papule	13.3	22.9	13.3	13.3 [4.3, 41.5]	1.2	4.3	3
Surgical and medical procedures	10061105	Dialysis	5.1	6.3	5.1	5.1 [1.7, 16]	0.8	1.7	3
Vascular disorders	10021097	Hypotension	3.4	37.1	3.4	3.5 [2.3, 5.3]	1.2	2.3	23
	10040560	Shock	18.0	224.7	18.0	18.4 [11, 30.6]	2.5	10.8	15
	10047115	Vasculitis	17.6	93.7	17.6	17.8 [8.4, 37.4]	2	8.4	7
	10011703	Cyanosis	9.3	29.2	9.3	9.4 [3.9, 22.5]	1.3	3.9	5

SOC: System organ classification; MedDRA: Medical dictionary of adverse events; PRR: Proportional reporting ratio; χ^2^: Chi-square test; RRR: Relative reporting ratio; ROR: Reporting odds ratio; IC: Information component; and EBGM: Empirical Bayes Geometric Mean.

**Table 7:** Signal detection measures for adverse events with tazobactam.

SOC	MedDRA	Adverse Event	PRR	χ^2^	RRR	ROR [95% CI]	IC025	EBGM05	Number of Cases
Blood and lymphatic system disorders	10016288	Febrile neutropenia	3.5	78.1	3.5	3.6 [2.7, 4.8]	1.4	2.6	44
	10018916	Hemolytic anemia	20.1	682.4	19.9	20.2 [14.7, 27.7]	3.1	14.5	39
	10001507	Agranulocytosis	10.6	318.7	10.5	10.6 [7.7, 14.7]	2.5	7.6	38
	10065553	Bone marrow failure	6.8	146.7	6.8	6.8 [4.8, 9.7]	2	4.7	31
	10028584	Myelosuppression	5.1	95.4	5.1	5.1 [3.6, 7.4]	1.6	3.6	30
	10009802	Coagulopathy	7.1	130.5	7.1	7.2 [4.9, 10.5]	1.9	4.8	26
	10071583	Hemophagocytic lymphohistiocytosis	16.3	299.1	16.2	16.4 [10.8, 24.9]	2.6	10.6	22
	10018910	Hemolysis	13.6	220.5	13.6	13.7 [8.8, 21.2]	2.4	8.7	20
	10013442	Disseminated intravascular coagulation	6.5	83.0	6.5	6.5 [4.2, 10.3]	1.7	4.1	19
	10073785	Autoimmune hemolytic anemia	24.4	354.9	24.2	24.5 [15.2, 39.5]	2.8	15	17
	10083842	Immune thrombocytopenia	14.8	152.4	14.7	14.8 [8.6, 25.5]	2.2	8.5	13
	10053873	Evans syndrome	77.6	368.6	75.2	77.7 [34.5, 175.3]	2.8	33.4	6
	10056335	Factor V inhibition	137.5	645.5	130.2	137.7 [60.5, 313.6]	3.7	57.1	6
	10073391	Platelet dysfunction	84.9	403.1	82.1	85.0 [37.7, 192.1]	2.8	36.3	6
	10075185	Allergic eosinophilia	859.6	2558.3	633.6	860.4 [309.8, 2389.6]	3.5	228	5
	10062713	Hemorrhagic diathesis	6.7	18.7	6.7	6.7 [2.8, 16.1]	1.1	2.8	5
	10050245	Autoimmune thrombocytopenia	32.7	92.1	32.3	32.8 [12.2, 87.9]	1.9	12	4
	10048930	Factor V deficiency	103.5	297.2	99.3	103.6 [38.1, 281.9]	2.4	36.5	4
	10043561	Thrombocytopenic purpura	13.2	33.6	13.2	13.2 [5, 35.4]	1.4	4.9	4
	10002046	Anemia hemolytic autoimmune	13.8	23.9	13.8	13.8 [4.4, 43.1]	1.2	4.4	3
	10061729	Bone marrow disorder	4.2	4.4	4.2	4.2 [1.3, 12.9]	0.7	1.3	3
	10021245	Idiopathic thrombocytopenic purpura	7.1	10.2	7.1	7.1 [2.3, 22.1]	0.9	2.3	3
	10025280	Lymphocytosis	6.3	8.6	6.3	6.3 [2, 19.7]	0.9	2	3
Cardiac disorders	10043071	Tachycardia	3.6	132.2	3.6	3.7 [2.9, 4.7]	1.5	2.9	70
	10007515	Cardiac arrest	3.1	84.0	3.1	3.2 [2.4, 4.1]	1.3	2.4	59
	10007617	Cardio-respiratory arrest	3.3	46.1	3.2	3.3 [2.3, 4.6]	1.2	2.3	31
	10044066	Torsade de pointes	8.3	94.5	8.2	8.3 [5.1, 13.5]	1.9	5	16
	10069167	Kounis syndrome	27.4	351.8	27.1	27.5 [16.5, 45.8]	2.9	16.3	15
	10058151	Pulseless electrical activity	12.6	105.8	12.6	12.6 [7, 22.9]	2	7	11
	10024119	Left ventricular failure	5.4	10.3	5.4	5.4 [2, 14.4]	0.9	2	4
	10007601	Cardiac pacemaker removal	200.6	381.0	185.2	200.7 [61.8, 651.9]	2.3	57	3
	10049434	Cardiac pacemaker replacement	30.3	57.6	30.0	30.4 [9.7, 94.8]	1.6	9.6	3
	10057651	Cardiac valve vegetation	27.7	52.1	27.4	27.7 [8.9, 86.4]	1.5	8.8	3
	10050011	Electromechanical dissociation	14.5	25.2	14.4	14.5 [4.7, 45.1]	1.2	4.6	3
	10024803	Long QT syndrome	5.3	6.6	5.3	5.3 [1.7, 16.4]	0.8	1.7	3
Eye disorders	10015993	Eyelid edema	5.3	44.4	5.3	5.3 [3.1, 9]	1.4	3.1	14
	10042690	Swelling of eyelid	4.3	9.5	4.3	4.3 [1.8, 10.3]	0.9	1.8	5
	10015683	Exophthalmos	6.5	8.9	6.5	6.5 [2.1, 20.1]	0.9	2.1	3
Gastrointestinal disorders	10024570	Lip swelling	4.0	63.9	4.0	4.0 [2.8, 5.7]	1.4	2.8	30
	10016100	Feces discolored	2.4	7.7	2.4	2.4 [1.3, 4.4]	0.7	1.3	11
	10082129	Dysbiosis	72.9	620.5	70.8	73.1 [38.9, 137.2]	3.3	37.7	10
	10027141	Melena	2.1	4.5	2.1	2.1 [1.1, 3.8]	0.6	1.1	10
	10057371	Hypoesthesia oral	2.4	5.1	2.4	2.4 [1.2, 4.8]	0.6	1.2	8
	10077605	Anal incontinence	3.1	5.0	3.0	3.1 [1.3, 7.3]	0.7	1.3	5
	10080276	Trichoglossia	53.2	202.3	52.1	53.3 [22, 129.3]	2.4	21.5	5
	10021333	Ileus paralytic	4.4	7.4	4.4	4.4 [1.7, 11.8]	0.8	1.6	4
	10022642	Intestinal dilatation	16.0	41.9	15.9	16.0 [6, 42.8]	1.5	5.9	4
	10022680	Intestinal ischemia	3.3	4.4	3.3	3.3 [1.2, 8.8]	0.6	1.2	4
	10024558	Lip edema	4.2	6.9	4.2	4.2 [1.6, 11.3]	0.8	1.6	4
	10027110	Megacolon	12.2	30.6	12.2	12.2 [4.6, 32.7]	1.3	4.6	4
	10028124	Mucosal ulceration	16.5	43.3	16.4	16.5 [6.2, 44]	1.5	6.1	4
General disorders and administration site conditions	10037660	Pyrexia	4.7	962.1	4.7	5.0 [4.4, 5.5]	2	4.2	327
	10010264	Condition aggravated	2.0	65.3	2.0	2.0 [1.7, 2.4]	0.8	1.7	129
	10008531	Chills	4.3	254.2	4.3	4.3 [3.6, 5.3]	1.7	3.5	103
	10008469	Chest discomfort	3.2	99.7	3.2	3.2 [2.5, 4.1]	1.3	2.5	67
	10051118	Drug ineffective for unapproved indication	4.8	180.1	4.7	4.8 [3.7, 6.2]	1.7	3.7	62
	10066901	Treatment failure	2.9	68.4	2.9	2.9 [2.2, 3.7]	1.2	2.2	58
	10059866	Drug resistance	8.8	376.2	8.7	8.8 [6.8, 11.5]	2.4	6.7	56
	10077361	Multiple organ dysfunction syndrome	7.5	216.7	7.4	7.5 [5.5, 10.2]	2.1	5.4	40
	10016029	Face edema	8.3	183.6	8.2	8.3 [5.8, 11.9]	2.1	5.7	30
	10057040	Temperature intolerance	11.6	239.5	11.5	11.6 [7.9, 17.1]	2.4	7.8	26
	10028154	Multi-organ failure	5.1	68.6	5.1	5.1 [3.4, 7.8]	1.5	3.4	22
	10020843	Hyperthermia	8.2	75.4	8.2	8.3 [4.8, 14.2]	1.8	4.8	13
	10020741	Hyperpyrexia	15.3	145.1	15.2	15.3 [8.7, 27]	2.2	8.6	12
	10015866	Extravasation	7.6	33.8	7.6	7.6 [3.6, 16]	1.4	3.6	7
	10018092	Generalized edema	2.8	6.5	2.8	2.8 [1.3, 5.9]	0.7	1.3	7
	10064774	Infusion site extravasation	6.2	25.4	6.2	6.2 [2.9, 13]	1.2	2.9	7
	10011912	Death neonatal	14.3	61.2	14.2	14.3 [6.4, 32]	1.7	6.3	6
	10048659	Concomitant disease progression	10.9	35.4	10.8	10.9 [4.5, 26.2]	1.4	4.5	5
	10053664	Infusion site pruritus	10.8	35.1	10.8	10.8 [4.5, 26.1]	1.4	4.5	5
	10068072	Potentiating drug interaction	6.9	19.6	6.9	6.9 [2.9, 16.6]	1.2	2.9	5
	10018691	Granuloma	5.5	10.4	5.5	5.5 [2, 14.6]	0.9	2	4
	10062654	Foaming at mouth	6.6	9.1	6.5	6.6 [2.1, 20.4]	0.9	2.1	3
Hepatobiliary disorders	10008635	Cholestasis	16.0	798.2	15.9	16.2 [12.5, 21]	3.1	12.3	58
	10019670	Hepatic function abnormal	7.3	308.1	7.3	7.4 [5.7, 9.5]	2.2	5.6	58
	10019837	Hepatocellular injury	14.9	501.7	14.8	15.0 [11, 20.5]	2.8	10.8	40
	10024690	Liver function test abnormal	5.4	135.0	5.4	5.4 [4, 7.4]	1.8	3.9	39
	10049199	Hepatic cytolysis	16.6	521.9	16.4	16.7 [12, 23]	2.9	11.9	37
	10072268	Drug-induced liver injury	5.1	109.4	5.1	5.2 [3.7, 7.3]	1.7	3.7	34
	10067125	Liver injury	5.9	118.3	5.9	6.0 [4.2, 8.6]	1.7	4.1	30
	10019717	Hepatitis	4.9	84.0	4.9	5.0 [3.4, 7.2]	1.6	3.4	28
	10019663	Hepatic failure	3.9	54.9	3.9	3.9 [2.7, 5.7]	1.3	3.7	27
	10024670	Liver disorder	2.6	23.2	2.6	2.6 [1.7, 3.8]	0.9	1.7	26
	10019851	Hepatotoxicity	4.2	53.5	4.2	4.2 [2.8, 6.4]	1.4	2.8	23
	10019754	Hepatitis cholestatic	15.7	231.1	15.6	15.7 [9.9, 25]	2.5	9.8	18
	10023126	Jaundice	2.6	14.7	2.6	2.6 [1.6, 4.3]	0.9	1.6	16
	10066758	Mixed liver injury	24.9	271.7	24.6	24.9 [14.4, 43.2]	2.7	14.3	13
	10067969	Cholestatic liver injury	22.2	180.5	22.0	22.3 [11.9, 41.6]	2.4	11.8	10
	10023129	Jaundice cholestatic	12.7	84.9	12.6	12.7 [6.6, 24.4]	1.9	6.5	9
	10008614	Cholecystitis acute	4.9	15.1	4.9	4.9 [2.2, 11]	1	2.2	6
	10019842	Hepatomegaly	2.8	5.3	2.8	2.8 [1.3, 6.3]	0.7	1.3	6
	10084058	Congestive hepatopathy	68.4	260.6	66.5	68.4 [28.1, 166.5]	2.5	27.4	5
	10019795	Hepatitis toxic	6.9	14.7	6.9	6.9 [2.6, 18.4]	1	2.6	4
	10050904	Cytolytic hepatitis	4.6	5.3	4.6	4.6 [1.5, 14.4]	0.7	1.5	3
Immune system disorders	10013700	Drug hypersensitivity	3.5	349.4	3.5	3.6 [3.1, 4.1]	1.6	3	196
	10002198	Anaphylactic reaction	9.6	876.9	9.6	9.8 [8.1, 11.8]	2.7	8	115
	10020751	Hypersensitivity	2.6	108.6	2.6	2.7 [2.2, 3.2]	1.2	2.2	108
	10002199	Anaphylactic shock	12.1	636.6	12.0	12.2 [9.5, 15.6]	2.8	9.4	64
	10023125	Jarisch–Herxheimer reaction	98.8	1488.8	95.0	99.1 [61, 161.2]	4	58.4	17
	10053613	Type IV hypersensitivity reaction	18.4	178.9	18.3	18.4 [10.4, 32.5]	2.4	10.3	12
	10048595	Histiocytosis hematophagic	13.6	103.7	13.5	13.6 [7.3, 25.3]	2	7.6	10
	10002216	Anaphylactoid reaction	8.7	47.0	8.7	8.7 [4.4, 17.5]	1.6	4.3	8
	10011411	Cross sensitivity reaction	35.9	167.8	35.4	36.0 [16.1, 80.6]	2.3	15.8	6
	10040400	Serum sickness	8.0	23.8	7.9	8.0 [3.3, 19.2]	1.2	3.3	5
	10040402	Serum-sickness-like reaction	35.6	133.4	35.1	35.6 [14.7, 86.2]	2.1	14.5	5
	10045240	Type I hypersensitivity	6.2	16.8	6.2	6.2 [2.6, 14.9]	1	2.6	5
Infections and infestations	10034133	Pathogen resistance	54.5	6166.2	53.3	55.8 [46.5, 66.9]	4.8	44.4	121
	10040047	Sepsis	3.8	170.4	3.8	3.8 [3.1, 4.7]	1.5	3	85
	10040070	Septic shock	8.9	500.6	8.8	9.0 [7.1, 11.3]	2.5	7	73
	10054236	Clostridium difficile infection	17.7	986.7	17.6	17.9 [14, 23]	3.2	13.7	64
	10009657	Clostridium difficile colitis	23.9	976.6	23.7	24.1 [18, 32.2]	3.4	17.7	46
	10017523	Fungemia	100.7	2936.4	96.7	101.3 [71, 144.4]	4.6	67.8	32
	10074170	Candida infection	7.6	124.6	7.6	7.6 [5.1, 11.5]	1.9	5	23
	10061043	Clostridial infection	22.7	431.5	22.5	22.8 [15, 34.8]	3	14.8	22
	10061471	Pseudomonas infection	13.5	228.6	13.4	13.5 [8.8, 20.8]	2.4	8.7	21
	10058080	Staphylococcal infection	2.8	20.0	2.8	2.8 [1.8, 4.4]	0.9	1.8	19
	10017533	Fungal infection	2.3	11.6	2.3	2.3 [1.5, 3.8]	0.8	1.4	17
	10061259	Klebsiella infection	18.3	259.6	18.2	18.4 [11.4, 29.7]	2.6	11.3	17
	10061124	Enterococcal infection	18.6	247.1	18.5	18.6 [11.4, 30.5]	2.6	11.3	16
	10053461	Trichosporon infection	184.3	2541.2	171.2	184.8 [111.1, 307.5]	4.5	102.9	16
	10003997	Bacteremia	6.6	66.1	6.6	6.6 [4, 11]	1.6	4	15
	10014665	Endocarditis	11.4	102.9	11.3	11.4 [6.5, 20.1]	2	6.4	12
	10060945	Bacterial infection	2.8	9.7	2.8	2.8 [1.5, 5.2]	0.8	1.5	10
	10064687	Device-related infection	3.3	12.1	3.3	3.3 [1.7, 6.3]	0.9	1.7	9
	10061126	Escherichia infection	5.1	22.2	5.1	5.1 [2.5, 10.2]	1.2	2.5	8
	10037128	Pseudomembranous colitis	18.0	111.4	17.9	18.1 [9, 36.2]	2.1	8.9	8
	10042938	Systemic candida	17.4	107.3	17.3	17.5 [8.7, 35]	2	8.5	8
	10056660	Geotrichum infection	189.3	1047.2	175.6	189.6 [87.8, 409.3]	3.5	81.3	7
	10060921	Abdominal abscess	7.4	27.2	7.4	7.4 [3.3, 16.6]	1.3	3.3	6
	10014666	Endocarditis bacterial	35.0	163.1	34.5	35.0 [15.6, 78.4]	2.3	15.4	6
	10054160	Klebsiella sepsis	29.2	134.7	28.8	29.2 [13.1, 65.4]	2.2	12.9	6
	10024652	Liver abscess	9.3	36.2	9.2	9.3 [4.2, 20.7]	1.4	4.1	6
	10028098	Mucormycosis	10.7	43.4	10.7	10.7 [4.8, 23.9]	1.5	4.8	6
	10061354	Pneumonia fungal	7.6	27.8	7.5	7.6 [3.4, 16.9]	1.3	3.4	6
	10035734	Pneumonia staphylococcal	16.1	70.2	16.0	16.2 [7.2, 36.1]	1.7	7.2	6
	10048709	Urosepsis	3.3	7.5	3.3	3.3 [1.5, 7.4]	0.8	1.5	6
	10006041	Botulism	37.6	141.2	37.0	37.6 [15.6, 91.1]	2.2	15.3	5
	10058305	Clostridium colitis	19.6	69.9	19.4	19.6 [8.1, 47.2]	1.8	8	5
	10015296	Escherichia sepsis	7.4	16.3	7.4	7.5 [2.8, 19.9]	1.1	2.8	4
	10071699	Infectious pleural effusion	13.6	34.6	13.5	13.6 [5.1, 36.3]	1.4	5	4
	10058806	Mycobacterium avium complex infection	7.1	15.2	7.0	7.1 [2.6, 18.9]	1.1	2.6	4
	10051017	Staphylococcal bacteremia	5.2	9.6	5.2	5.2 [1.9, 13.8]	0.9	1.9	4
	10001980	Amoebiasis	58.7	114.5	57.3	58.7 [18.7, 184.7]	1.9	18.2	3
	10001985	Amoebic colitis	120.3	234.0	114.7	120.4 [37.8, 384]	2.1	36	3
	10007810	Catheter-related infection	11.0	18.1	10.9	11.0 [3.5, 34.1]	1.1	3.5	3
	10058666	Cytomegalovirus infection reactivation	4.8	5.6	4.8	4.8 [1.5, 14.9]	0.8	1.6	3
	10012742	Diarrhea infectious	20.7	37.9	20.5	20.7 [6.6, 64.5]	1.4	6.6	3
	10014568	Empyema	7.5	11.0	7.5	7.5 [2.4, 23.3]	0.9	2.4	3
	10051998	Febrile infection	15.0	26.2	14.9	15.0 [4.8, 46.7]	1.3	4.8	3
	10020431	Human herpesvirus 6 infection	6.9	9.8	6.9	6.9 [2.2, 21.5]	0.9	2.2	3
	10021784	Infected skin ulcer	7.9	11.8	7.9	7.9 [2.5, 24.6]	1	2.5	3
	10060738	Intervertebral discitis	8.9	13.9	8.9	8.9 [2.9, 27.8]	1	2.9	3
	10058923	Pseudomonal bacteremia	20.4	37.3	20.2	20.4 [6.5, 63.6]	1.4	6.5	3
	10070087	Raoultella ornithinolytica infection	225.6	425.2	206.4	225.8 [69.1, 737.5]	2.4	63.2	3
	10047931	Whipple’s disease	34.5	66.1	34.1	34.6 [11.1, 108.1]	1.6	10.9	3
Injury, poisoning, and procedural complications	10076476	Product use in unapproved indication	2.1	64.6	2.1	2.1 [1.8, 2.5]	0.9	1.7	115
	10051792	Infusion-related reaction	2.5	20.9	2.5	2.5 [1.7, 3.8]	0.9	1.7	24
	10073311	Occupational exposure to product	3.4	9.5	3.4	3.4 [1.6, 7.1]	0.7	1.6	7
	10064372	Documented hypersensitivity to administered drug	72.9	346.3	70.8	73.0 [32.4, 164.6]	2.7	31.4	6
	10064306	Incorrect drug administration rate	7.8	29.1	7.8	7.8 [3.5, 17.5]	1.3	3.5	6
	10076470	Documented hypersensitivity to administered product	27.0	99.6	26.8	27.1 [11.2, 65.4]	2	11	5
	10075462	Drug monitoring procedure not performed	9.0	20.9	9.0	9.0 [3.4, 24]	1.2	3.4	4
	10084721	Labeled drug–drug interaction issue	11.9	19.9	11.8	11.9 [3.8, 37]	1.1	3.8	3
	10051373	Wound hemorrhage	4.0	4.1	4.0	4.0 [1.3, 12.4]	0.6	1.3	3
Investigations	10043554	Thrombocytopenia	8.7	1350.8	8.6	9.0 [7.8, 10.3]	2.7	7.5	200
	10029354	Neutropenia	3.6	201.0	3.6	3.6 [3, 4.4]	1.5	3	109
	10014950	Eosinophilia	23.8	1771.0	23.5	24.1 [19.4, 30]	3.7	18.9	83
	10047942	White blood cell count decreased	3.4	130.3	3.4	3.4 [2.7, 4.2]	1.4	2.7	80
	10005483	Blood creatinine increased	5.6	297.2	5.6	5.7 [4.6, 7.1]	2	4.5	79
	10035528	Platelet count decreased	3.3	121.4	3.3	3.4 [2.7, 4.2]	1.4	2.6	76
	10001551	Alanine aminotransferase increased	5.4	249.8	5.4	5.5 [4.4, 7]	1.9	4.3	70
	10070027	Clostridium test positive	166.9	10021.8	156.1	169.0 [131.5, 217.2]	5.7	121.5	66
	10024384	Leukopenia	6.4	291.2	6.4	6.5 [5.1, 8.3]	2.1	5	65
	10033318	Oxygen saturation decreased	5.9	229.0	5.9	5.9 [4.6, 7.7]	2	4.5	58
	10003481	Aspartate aminotransferase increased	4.9	165.8	4.9	5.0 [3.8, 6.5]	1.8	3.8	54
	10033661	Pancytopenia	4.7	155.8	4.7	4.8 [3.7, 6.3]	1.7	3.6	54
	10005734	Blood pressure decreased	2.9	49.8	2.9	2.9 [2.2, 4]	1.1	2.1	41
	10006825	C-reactive protein increased	5.9	144.5	5.9	5.9 [4.3, 8.2]	1.8	4.2	37
	10060795	Hepatic enzyme increased	2.5	31.6	2.5	2.5 [1.8, 3.5]	1	1.8	36
	10054889	Transaminases increased	6.4	134.7	6.4	6.4 [4.5, 9.1]	1.9	4.5	31
	10059570	Blood alkaline phosphatase increased	5.2	88.5	5.2	5.3 [3.6, 7.7]	1.6	3.6	27
	10005911	Body temperature increased	6.5	119.5	6.5	6.5 [4.5, 9.5]	1.8	4.4	27
	10021015	Hypokalemia	2.8	27.3	2.8	2.8 [1.9, 4.2]	1	1.9	25
	10029366	Neutrophil count decreased	3.0	30.9	3.0	3.0 [2, 4.4]	1.1	2	25
	10047943	White blood cell count increased	3.2	35.6	3.2	3.2 [2.2, 4.8]	1.1	2.2	25
	10022595	International normalized ratio increased	3.4	37.0	3.4	3.4 [2.3, 5.2]	1.2	2.3	23
	10024378	Leukocytosis	6.0	91.8	6.0	6.1 [4, 9.1]	1.7	4	23
	10017693	Gamma-glutamyl transferase increased	5.1	69.0	5.1	5.1 [3.4, 7.8]	1.5	3.4	22
	10005364	Blood bilirubin increased	3.6	36.9	3.6	3.6 [2.4, 5.5]	1.2	2.3	21
	10020578	Hyperbilirubinemia	10.2	164.3	10.1	10.2 [6.7, 15.7]	2.2	6.6	21
	10014945	Eosinophil count increased	11.3	177.1	11.3	11.4 [7.3, 17.6]	2.2	7.3	20
	10020679	Hypernatremia	20.9	355.8	20.7	21.0 [13.5, 32.6]	2.8	13.3	20
	10005630	Blood lactate dehydrogenase increased	5.6	60.0	5.6	5.6 [3.5, 9.1]	1.5	3.5	17
	10013722	Drug level increased	3.0	14.1	3.0	3.0 [1.7, 5.3]	0.9	1.7	12
	10005851	Blood urea increased	3.1	12.0	3.1	3.1 [1.7, 5.7]	0.9	1.6	10
	10069826	Inflammatory marker increased	12.4	93.5	12.4	12.4 [6.7, 23.2]	1.9	6.6	10
	10025327	Lymphopenia	3.4	14.5	3.4	3.4 [1.8, 6.3]	0.9	1.8	10
	10000636	Activated partial thromboplastin time prolonged	8.0	48.3	8.0	8.0 [4.2, 15.4]	1.6	4.1	9
	10018838	Hematocrit decreased	2.2	5.0	2.2	2.2 [1.2, 4.3]	0.6	1.2	9
	10037063	Prothrombin time prolonged	6.7	38.0	6.7	6.7 [3.5, 12.9]	1.4	3.5	9
	10040250	Serum ferritin increased	7.9	41.3	7.9	7.9 [3.9, 15.8]	1.5	3.9	8
	10058956	Bicytopenia	20.1	107.7	19.9	20.1 [9.6, 42.3]	2.1	9.5	7
	10018681	Granulocyte count decreased	20.2	108.1	20.0	20.2 [9.6, 42.5]	2.1	9.5	7
	10051608	Platelet count increased	2.4	4.3	2.4	2.4 [1.1, 5]	0.6	1.1	7
	10070052	Staphylococcus test positive	18.9	100.5	18.7	18.9 [9, 39.8]	2	8.9	7
	10005803	Blood sodium increased	10.8	44.0	10.8	10.8 [4.9, 24.2]	1.5	4.8	6
	10068237	Hypertransaminasemia	4.8	14.4	4.8	4.8 [2.2, 10.7]	1	2.1	6
	10020942	Hypoalbuminemia	4.4	12.7	4.4	4.4 [2, 9.9]	1	2	6
	10043563	Thrombocytosis	7.8	28.9	7.8	7.8 [3.5, 17.3]	1.3	3.5	6
	10005488	Blood culture positive	7.7	22.7	7.7	7.7 [3.2, 18.5]	1.2	3.2	5
	10058363	Eosinophils urine present	388.2	1346.0	334.4	388.6 [151, 999.7]	3.3	130	5
	10024574	Lipase increased	3.0	4.8	3.0	3.0 [1.3, 7.2]	0.7	1.2	5
	10067081	Procalcitonin increased	24.0	87.6	23.8	24.0 [10, 58]	1.9	9.9	5
	10037469	Pulse absent	4.7	11.2	4.7	4.7 [2, 11.4]	0.9	2	5
	10059895	Urine output decreased	2.9	4.5	2.9	2.9 [1.2, 7]	0.6	1.2	5
	10002793	Antimicrobial susceptibility test resistant	46.7	133.6	45.9	46.8 [17.4, 125.8]	2.1	17	4
	10014391	Electrocardiogram ST segment depression	8.3	18.8	8.3	8.3 [3.1, 22.1]	1.1	3.1	4
	10072234	Enzyme level increased	21.3	57.8	21.1	21.3 [7.9, 56.9]	1.6	7.9	4
	10069962	Acinetobacter test positive	75.2	147.1	73.0	75.3 [23.8, 237.5]	2	23.1	3
	10001549	Alanine aminotransferase decreased	23.5	43.7	23.3	23.5 [7.5, 73.4]	1.5	7.5	3
	10058542	Anti-platelet antibody	328.2	597.2	288.9	328.4 [98.3, 1097.5]	2.4	86.5	3
	10049471	Blood phosphorus decreased	4.0	4.0	4.0	4.0 [1.3, 12.3]	0.6	1.3	3
	10050380	Electrocardiogram t wave abnormal	8.5	12.9	8.4	8.5 [2.7, 26.3]	1	2.7	3
	10070090	Escherichia test positive	14.3	24.8	14.2	14.3 [4.6, 44.5]	1.2	4.6	3
	10070748	False positive investigation result	10.9	18.0	10.9	10.9 [3.5, 34]	1.1	3.5	3
	10067350	Hypercreatinemia	46.9	91.0	46.0	46.9 [15, 147.1]	1.8	14.7	3
	10070091	Klebsiella test positive	26.4	49.7	26.2	26.5 [8.5, 82.6]	1.5	8.4	3
	10061313	Neutrophil count abnormal	4.9	5.7	4.9	4.9 [1.6, 15.1]	0.7	1.6	3
	10061880	Occult blood positive	6.6	9.2	6.6	6.6 [2.1, 20.5]	0.9	2.1	3
	10059168	Oxygen consumption increased	6.3	8.5	6.2	6.3 [2, 19.5]	0.9	2	3
	10037070	Prothrombin time shortened	10.3	16.7	10.3	10.3 [3.3, 32.1]	1.1	3.3	3
Metabolism and nutrition disorders	10027417	Metabolic acidosis	2.9	25.4	2.9	2.9 [1.9, 4.4]	1	1.9	22
	10016803	Fluid overload	2.6	4.4	2.6	2.6 [1.2, 5.8]	0.6	1.2	6
	10047634	Vitamin K deficiency	41.0	79.2	40.4	41.0 [13.1, 128.5]	1.7	12.9	3
Musculoskeletal and connective tissue disorders	10039020	Rhabdomyolysis	2.1	11.1	2.1	2.1 [1.4, 3.2]	0.7	1.4	21
	10021119	Hypotonia neonatal	6.8	9.7	6.8	6.8 [2.2, 21.3]	0.9	2.2	3
Nervous system disorders	10014625	Encephalopathy	6.8	171.2	6.8	6.8 [4.9, 9.5]	2	4.9	36
	10012218	Delirium	3.2	33.7	3.2	3.2 [2.1, 4.8]	1.1	2.1	24
	10012373	Depressed level of consciousness	2.0	9.1	2.0	2.0 [1.3, 3.2]	0.7	1.3	19
	10044221	Toxic encephalopathy	13.2	145.0	13.1	13.2 [7.8, 22.4]	2.2	7.8	14
	10045555	Unresponsive to stimuli	2.5	10.8	2.5	2.5 [1.5, 4.2]	0.8	1.5	14
	10028622	Myoclonus	4.5	31.8	4.5	4.5 [2.6, 7.8]	1.3	4.6	13
	10029350	Neurotoxicity	3.3	16.7	3.3	3.3 [1.9, 5.8]	1	1.9	12
	10003547	Asterixis	16.5	43.6	16.4	16.6 [6.2, 44.3]	1.5	6.1	4
	10061666	Autonomic neuropathy	13.9	24.1	13.8	13.9 [4.5, 43.3]	1.2	4.5	3
	10022840	Intraventricular hemorrhage	5.0	6.0	5.0	5.0 [1.6, 15.5]	0.7	1.6	3
Pregnancy, puerperium, and perinatal conditions	10036590	Premature baby	2.2	10.9	2.2	2.2 [1.4, 3.4]	0.7	1.4	19
	10067508	Low birth weight baby	3.7	13.4	3.7	3.7 [1.9, 7.5]	0.9	1.8	8
	10071409	Fetal exposure during delivery	14.7	25.7	14.7	14.7 [4.7, 45.9]	1.2	4.7	3
	10071407	Maternal exposure during delivery	15.7	27.7	15.6	15.7 [5, 48.9]	1.3	5	3
Product issues	10069250	Product deposit	31.3	260.8	30.9	31.4 [16.8, 58.6]	2.7	16.6	10
	10069293	Product container issue	4.4	20.6	4.4	4.4 [2.3, 8.6]	1.1	2.3	9
	10064685	Device occlusion	2.6	4.2	2.6	2.6 [1.1, 5.7]	0.6	1.1	6
	10070617	Device infusion issue	12.4	41.2	12.3	12.4 [5.1, 29.8]	1.5	5.1	5
	10081540	Recalled product administered	3.0	4.8	3.0	3.0 [1.2, 7.2]	0.7	1.2	5
Psychiatric disorders	10013954	Dysphoria	4.7	17.1	4.7	4.8 [2.3, 10]	1.1	2.3	7
	10076227	Disorganized speech	9.7	23.1	9.7	9.7 [3.6, 26]	1.2	3.6	4
Renal and urinary disorders	10069339	Acute kidney injury	5.0	541.9	5.0	5.1 [4.4, 6]	2	4.3	171
	10048302	Tubulointerstitial nephritis	20.7	1744.1	20.5	21.0 [17.1, 25.8]	3.6	16.7	95
	10038436	Renal failure acute	9.2	611.7	9.2	9.3 [7.5, 11.6]	2.6	7.4	85
	10062237	Renal impairment	4.1	182.2	4.1	4.1 [3.3, 5.2]	1.6	3.3	79
	10038435	Renal failure	2.2	43.8	2.2	2.2 [1.7, 2.7]	0.9	1.7	72
	10038540	Renal tubular necrosis	20.2	704.6	20.0	20.3 [14.9, 27.8]	3.2	14.7	40
	10029155	Nephropathy toxic	13.2	345.7	13.1	13.2 [9.3, 18.8]	2.6	9.2	32
	10024515	Linear IgA disease	71.3	1951.4	69.3	71.7 [49.8, 103.2]	4.2	48.1	30
	10029134	Nephritis interstitial	85.1	2167.0	82.2	85.5 [58.6, 124.8]	4.4	56.3	28
	10029117	Nephritis	29.0	507.0	28.7	29.1 [18.7, 45.3]	3.1	18.4	20
	10002847	Anuria	7.5	71.7	7.4	7.5 [4.4, 12.6]	1.7	4.4	14
	10061105	Dialysis	3.8	25.8	3.8	3.8 [2.2, 6.4]	1.1	2.2	14
	10018875	Hemodialysis	6.6	60.8	6.6	6.6 [3.9, 11.2]	1.6	3.9	14
	10029151	Nephropathy	3.5	11.7	3.5	3.5 [1.7, 7]	0.9	1.7	8
	10030302	Oliguria	6.1	29.4	6.1	6.1 [3.1, 12.3]	1.3	3.1	8
	10029147	Nephrogenic diabetes insipidus	23.6	128.5	23.4	23.6 [11.2, 49.8]	2.2	11.1	7
	10038537	Renal tubular disorder	8.3	25.2	8.3	8.3 [3.5, 20.1]	1.3	3.4	5
	10018906	Hemoglobinuria	17.2	45.6	17.1	17.2 [6.4, 46.1]	1.5	6.4	4
	10050791	Leukocyturia	19.6	35.6	19.4	19.6 [6.3, 61]	1.4	6.2	3
	10037686	Pyuria	14.6	25.5	14.5	14.6 [4.7, 45.5]	1.2	4.7	3
Respiratory, thoracic, and mediastinal disorders	10038695	Respiratory failure	5.5	308.0	5.5	5.6 [4.5, 6.9]	2	4.4	85
	10043089	Tachypnea	18.3	845.7	18.2	18.5 [14.1, 24.3]	3.2	13.9	53
	10047924	Wheezing	2.9	39.8	2.9	2.9 [2.1, 4.1]	1.1	2.1	33
	10021143	Hypoxia	4.5	80.2	4.5	4.5 [3.2, 6.4]	1.5	3.1	31
	10022611	Interstitial lung disease	2.8	30.2	2.8	2.8 [1.9, 4]	1	1.9	28
	10035669	Pneumonia aspiration	4.9	74.8	4.9	5.0 [3.4, 7.4]	1.6	3.3	25
	10001052	Acute respiratory distress syndrome	6.1	98.1	6.1	6.2 [4.1, 9.2]	1.8	4.1	24
	10038687	Respiratory distress	3.6	36.9	3.6	3.6 [2.4, 5.5]	1.1	2.3	21
	10038712	Respiratory rate increased	11.3	176.2	11.2	11.3 [7.3, 17.6]	2.2	7.2	20
	10023845	Laryngeal edema	14.3	221.0	14.2	14.4 [9.1, 22.6]	2.4	9	19
	10006482	Bronchospasm	6.0	65.3	6.0	6.0 [3.7, 9.6]	1.6	3.7	17
	10043528	Throat tightness	2.5	12.5	2.5	2.5 [1.5, 4.2]	0.8	1.5	15
	10006473	Bronchopulmonary aspergillosis	9.1	93.2	9.1	9.2 [5.4, 15.5]	1.9	5.4	14
	10018964	Hemoptysis	2.5	10.7	2.5	2.5 [1.5, 4.2]	0.8	1.5	14
	10025102	Lung infiltration	5.2	30.0	5.2	5.2 [2.8, 9.7]	1.3	2.8	10
	10035731	Pneumonia pseudomonal	41.6	351.0	40.9	41.7 [22.3, 78]	2.9	21.9	10
	10052832	Eosinophilic pneumonia acute	47.3	355.9	46.4	47.4 [24.5, 91.7]	2.8	24	9
	10064780	Breath sounds abnormal	8.2	37.1	8.2	8.2 [3.9, 17.2]	1.4	3.9	7
	10037833	Rales	6.7	28.3	6.7	6.7 [3.2, 14]	1.3	3.2	7
	10003497	Asphyxia	3.2	6.9	3.2	3.2 [1.4, 7.1]	0.7	1.4	6
	10003598	Atelectasis	3.4	7.9	3.4	3.4 [1.5, 7.6]	0.8	1.5	6
	10037382	Pulmonary eosinophilia	48.6	229.6	47.7	48.7 [21.7, 109.3]	2.5	21.2	6
	10038661	Respiratory acidosis	7.0	24.9	6.9	7.0 [3.1, 15.5]	1.3	3.1	6
	10039109	Rhonchi	25.6	116.8	25.3	25.6 [11.4, 57.2]	2.1	11.3	6
	10042241	Stridor	9.6	38.0	9.6	9.6 [4.3, 21.5]	1.4	4.3	6
	10001029	Acute pulmonary edema	4.8	11.3	4.8	4.8 [2, 11.5]	0.9	2	5
	10014962	Eosinophilic pneumonia	7.3	21.1	7.3	7.3 [3, 17.5]	1.2	3	5
	10038647	Respiration abnormal	3.3	5.9	3.3	3.3 [1.4, 8]	0.7	1.4	5
	10011376	Crepitations	6.4	13.3	6.4	6.4 [2.4, 17.2]	1	2.4	4
	10019027	Hemothorax	4.7	5.4	4.7	4.7 [1.5, 14.5]	0.7	1.5	3
	10020591	Hypercapnia	5.3	6.5	5.3	5.3 [1.7, 16.3]	0.8	1.7	3
	10021133	Hypoventilation	4.0	4.2	4.0	4.0 [1.3, 12.6]	0.6	1.3	3
	10025080	Lung consolidation	5.6	7.1	5.6	5.6 [1.8, 17.3]	0.8	1.8	3
	10028923	Neonatal asphyxia	16.3	29.0	16.2	16.3 [5.2, 50.9]	1.3	5.2	3
	10035717	Pneumonia Klebsiella	8.9	13.8	8.9	8.9 [2.9, 27.6]	1	2.8	3
	10070774	Respiratory tract edema	20.7	38.0	20.6	20.8 [6.7, 64.7]	1.4	6.6	3
	10042444	Suffocation feeling	5.9	7.8	5.9	5.9 [1.9, 18.4]	0.8	1.9	3
Skin and subcutaneous tissue disorders	10037844	Rash	3.7	688.7	3.7	3.9 [3.5, 4.3]	1.7	3.3	346
	10037087	Pruritus	3.1	348.7	3.1	3.2 [2.8, 3.7]	1.4	2.8	237
	10015150	Erythema	4.3	373.6	4.3	4.4 [3.7, 5.2]	1.8	3.7	147
	10073508	Drug reaction with eosinophilia and systemic symptoms	22.5	2813.9	22.3	23.1 [19.5, 27.4]	3.8	18.8	139
	10037855	Rash erythematous	10.0	686.6	10.0	10.2 [8.2, 12.6]	2.7	8.1	86
	10037868	Rash maculo-papular	19.1	1431.1	19.0	19.4 [15.7, 24.1]	3.4	15.3	85
	10046735	Urticaria	2.4	66.6	2.4	2.4 [1.9, 3]	1	1.9	85
	10002424	Angioedema	5.8	258.1	5.8	5.9 [4.6, 7.5]	2	4.5	66
	10013687	Drug eruption	17.8	975.1	17.7	18.0 [14, 23.1]	3.2	13.8	63
	10044223	Toxic epidermal necrolysis	14.2	570.8	14.1	14.3 [10.7, 19]	2.9	10.6	48
	10042033	Stevens–Johnson syndrome	7.4	232.6	7.4	7.5 [5.5, 10.1]	2.1	5.5	43
	10037884	Rash pruritic	3.7	74.4	3.6	3.7 [2.7, 5]	1.4	2.7	40
	10048799	Acute generalized exanthematous pustulosis	20.8	672.7	20.6	21.0 [15.1, 29]	3.2	14.9	37
	10057970	Toxic skin eruption	20.1	648.1	20.0	20.3 [14.6, 28]	3.1	14.4	37
	10005191	Blister	2.8	33.9	2.8	2.8 [2, 4]	1	2	31
	10034754	Petechiae	13.7	327.7	13.7	13.8 [9.6, 19.9]	2.6	9.5	29
	10040844	Skin exfoliation	2.3	20.7	2.3	2.3 [1.6, 3.4]	0.8	1.6	29
	10037858	Rash generalized	4.4	69.7	4.4	4.4 [3, 6.4]	1.5	3	28
	10012434	Dermatitis allergic	8.1	123.8	8.1	8.2 [5.3, 12.5]	2	5.3	21
	10037867	Rash macular	3.4	32.6	3.4	3.4 [2.2, 5.2]	1.1	2.2	21
	10037870	Rash morbilliform	32.6	544.2	32.2	32.8 [20.8, 51.6]	3.2	20.5	19
	10058919	Drug rash with eosinophilia and systemic symptoms	15.3	171.6	15.2	15.3 [9, 25.9]	2.3	9	14
	10040914	Skin reaction	3.6	18.0	3.6	3.6 [2, 6.5]	1	2	11
	10012431	Dermatitis	2.7	9.0	2.7	2.7 [1.4, 5]	0.8	1.4	10
	10012455	Dermatitis exfoliative	9.0	63.2	9.0	9.0 [4.8, 16.8]	1.7	4.8	10
	10037876	Rash papular	2.8	9.6	2.8	2.8 [1.5, 5.2]	0.8	1.5	10
	10078325	Symmetrical drug-related intertriginous and flexural exanthema	53.2	451.8	52.1	53.3 [28.5, 99.9]	3	27.8	10
	10033733	Papule	6.2	34.0	6.2	6.2 [3.2, 11.9]	1.4	3.2	9
	10037888	Rash pustular	5.9	32.1	5.9	5.9 [3.1, 11.4]	1.3	3.1	9
	10051576	Generalized erythema	6.5	31.9	6.5	6.5 [3.3, 13]	1.3	3.2	8
	10020764	Hypersensitivity vasculitis	11.2	55.1	11.2	11.2 [5.3, 23.6]	1.7	5.3	7
	10015218	Erythema multiforme	2.8	5.3	2.8	2.8 [1.3, 6.3]	0.7	1.3	6
	10016741	Fixed eruption	14.9	64.4	14.9	15.0 [6.7, 33.4]	1.7	6.7	6
	10064579	Exfoliative rash	9.4	29.5	9.4	9.4 [3.9, 22.7]	1.3	3.9	5
	10018999	Hemorrhage subcutaneous	6.2	16.8	6.2	6.2 [2.6, 14.9]	1.1	2.6	5
	10012456	Dermatitis exfoliative generalized	4.3	4.7	4.3	4.3 [1.4, 13.5]	0.7	1.4	3
	10048768	Dermatosis	21.8	40.2	21.6	21.8 [7, 68]	1.4	6.9	3
	10084905	Generalized bullous fixed drug eruption	44.6	86.3	43.8	44.6 [14.2, 139.8]	1.7	14	3
	10024377	Leukocytoclastic vasculitis	7.2	10.5	7.2	7.2 [2.3, 22.5]	0.9	2.3	3
	10025421	Macule	4.3	4.6	4.3	4.3 [1.4, 13.3]	0.7	1.4	3
Surgical and medical procedures	10059513	Palliative care	11.2	27.4	11.1	11.2 [4.2, 29.9]	1.3	4.2	4
Vascular disorders	10021097	Hypotension	2.8	137.7	2.8	2.8 [2.4, 3.4]	1.2	2.3	120
	10016825	Flushing	2.0	23.8	2.0	2.1 [1.5, 2.8]	0.8	1.5	46
	10011703	Cyanosis	7.9	156.6	7.9	8.0 [5.5, 11.7]	2	5.4	27
	10040560	Shock	4.3	55.2	4.3	4.3 [2.9, 6.5]	1.4	2.9	23
	10033546	Pallor	2.3	8.9	2.3	2.3 [1.4, 3.9]	0.7	1.4	14
	10037549	Purpura	8.0	79.2	8.0	8.1 [4.8, 13.6]	1.8	4.7	14
	10011686	Cutaneous vasculitis	10.9	61.9	10.8	10.9 [5.4, 21.8]	1.7	5.4	8
	10020565	Hyperemia	18.8	117.0	18.7	18.9 [9.4, 37.9]	2.1	9.3	8
	10047115	Vasculitis	3.2	9.7	3.2	3.2 [1.6, 6.3]	0.8	1.5	8
	10052076	Hemodynamic instability	3.6	8.6	3.6	3.6 [1.6, 8]	0.8	1.6	6
	10058017	Infective aneurysm	51.2	194.3	50.2	51.3 [21.1, 124.3]	2.3	20.7	5
	10034879	Phlebitis	4.9	11.7	4.9	4.9 [2, 11.7]	0.9	2	5
	10047097	Vascular purpura	11.8	19.7	11.7	11.8 [3.8, 36.7]	1.1	3.8	3

SOC: System organ classification; MedDRA: Medical dictionary of adverse events; PRR: Proportional reporting ratio; χ^2^: Chi-square test; RRR: Relative reporting ratio; ROR: Reporting odds ratio; IC: Information component; and EBGM: Empirical Bayes Geometric Mean.

**Table 8:** Signal detection measures for adverse events with vaborbactam.

SOC	MedDRA	Adverse Event	PRR	χ^2^	RRR	ROR	IC025	EBGM05	Number of Cases
General disorders and administration site conditions	10051118	Drug ineffective for unapproved indication	71.7	282.3	71.7	87.1 [33.1, 229.2]	2.3	27.3	5
Injury, poisoning, and procedural complications	10053762	Off label use	11.8	112.0	11.8	19.9 [9.4, 42]	1.7	5.6	12

SOC: System organ classification; MedDRA: Medical dictionary of adverse events; PRR: Proportional reporting ratio; χ^2^: Chi-square test; RRR: Relative reporting ratio; ROR: Reporting odds ratio; IC: Information component; and EBGM: Empirical Bayes Geometric Mean.

## Data Availability

The data supporting the findings of this study are available from the corresponding author upon reasonable request, as the authors did not seek permission to publicly share them from the data owner.
